# Extracellular Vesicles in Infrapatellar Fat Pad from Osteoarthritis Patients Impair Cartilage Metabolism and Induce Senescence

**DOI:** 10.1002/advs.202303614

**Published:** 2023-11-30

**Authors:** Yumei Cao, Jianzhao Ruan, Jingliang Kang, Xiaoyu Nie, Weiren Lan, Guangfeng Ruan, Jia Li, Zhaohua Zhu, Weiyu Han, Su'an Tang, Changhai Ding

**Affiliations:** ^1^ Clinical Research Centre Zhujiang Hospital Southern Medical University Guangzhou Guangdong 510260 China; ^2^ Department of Rheumatology and Immunology Zhujiang Hospital Southern Medical University Guangzhou Guangdong 510260 China; ^3^ Clinical Research Centre Guangzhou First People's Hospital School of Medicine South China University of Technology Guangzhou 510180 China; ^4^ Division of Orthopaedic Surgery Department of Orthopedics Nanfang Hospital Southern Medical University Guangzhou Guangdong 510515 China; ^5^ Centre of Orthopedics Zhujiang Hospital Southern Medical University Guangzhou Guangdong 510260 China; ^6^ Menzies Institute for Medical Research University of Tasmania Hobart Tasmania 7000 Australia

**Keywords:** extracellular vesicles, infrapatellar fat pad, lamin B receptor, osteoarthritis, senescence

## Abstract

Infrapatellar fat pad (IPFP) is closely associated with the development and progression of knee osteoarthritis (OA), but the underlying mechanism remains unclear. Here, it is find that IPFP from OA patients can secret small extracellular vesicles (sEVs) and deliver them into articular chondrocytes. Inhibition the release of endogenous osteoarthritic IPFP‐sEVs by GW4869 significantly alleviated IPFP‐sEVs‐induced cartilage destruction. Functional assays in vitro demonstrated that IPFP‐sEVs significantly promoted chondrocyte extracellular matrix (ECM) catabolism and induced cellular senescence. It is further demonstrated that IPFP‐sEVs induced ECM degradation in human and mice cartilage explants and aggravated the progression of experimental OA in mice. Mechanistically, highly enriched let‐7b‐5p and let‐7c‐5p in IPFP‐sEVs are essential to mediate detrimental effects by directly decreasing senescence negative regulator, lamin B receptor (LBR). Notably, intra‐articular injection of antagomirs inhibiting let‐7b‐5p and let‐7c‐5p in mice increased LBR expression, suppressed chondrocyte senescence and ameliorated the progression of experimental OA model. This study uncovers the function and mechanism of the IPFP‐sEVs in the progression of OA. Targeting IPFP‐sEVs cargoes of let‐7b‐5p and let‐7c‐5p can provide a potential strategy for OA therapy.

## Graphical abstract

Infrapatellar fat pad (IPFP) from knee osteoarthritis (OA) patients can secrete small extracellular vesicles (sEVs) and deliver them into articular chondrocytes. IPFP‐sEVs can promote chondrocyte catabolism and cellular senescence. Highly enriched let‐7b‐5p and let‐7c‐5p in IPFP‐sEVs are essential to mediate detrimental effects by directly decreasing senescence negative regulator, lamin B receptor (LBR). This study uncovers the function and mechanism of the IPFP‐sEVs in the progression of OA.

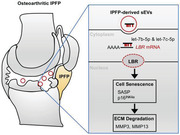

## 1 Introduction

Osteoarthritis (OA) is one of the most common degenerative joint diseases among elderly individuals, which is primarily characterized by cartilage destruction, synovial inflammation, aberrant mineralization in the subchondral bone and bony overgrowth in the form of osteophytes.^[^ ^1^, ^2^ ^]^ Despite many risk factors identified, including aging, obesity, trauma and genetics, the pathogenic mechanism of OA remains elusive.^[^ ^3^, ^4^ ^]^ Infrapatellar fat pad (IPFP) is the main adipose tissue within the knee joint, and capable of distributing mechanical force through the articular joint.^[^ ^5^, ^6^ ^]^ IPFP is involved in OA progression by secreting multiple proinflammatory cytokines and adipokines contributing to intra‐articular inflammation and joint pain.^[^ ^7^, ^8^, ^9^ ^]^ However, the molecular mechanism of IPFP affecting OA progression is not clear. Thus, characterizing the molecular mechanisms of chondrocytes involved in OA development and progression is crucial for developing diagnostic biomarkers and effective prevention strategies.

Small extracellular vesicles (sEVs), also referred as exosomes, are nano‐sized membrane‐bound vesicles with a size range of 30–200 nm in diameter.^[^ ^10^, ^11^ ^]^ Recent literature have termed “sEVs” instead of “exosomes” to represent EVs of size less than 200 nm, as recommended by minimal information for studies of Extracellular vesicles 2018 (MISEV2018).^[^ ^12^, ^13^ ^]^ These sEVs are critical intercellular and interorgan communicators encapsulating a variety of biologically active contents such as proteins, lipid, mRNAs, and noncoding RNAs.^[^ ^14^, ^15^ ^]^ Among the sEVs‐carrying cargoes, microRNAs (miRNAs) have attracted much attention due to their extensive and important roles.^[^ ^16^ ^]^ sEVs‐derived miRNAs could be taken up by adjacent cells or enter the circulation and reach distant sites, subsequently regulating downstream signaling by repressing target mRNAs in recipient cells.^[^ ^17^, ^18^ ^]^ Recent studies have shown that sEVs play key roles in OA progression. sEVs derived from IL‐1β‐stimulated human synovial fibroblasts and M1‐like macrophages could accelerate OA progression by sEVs‐miRNAs mediating extracellular matrix (ECM) metabolism.^[^ ^19^, ^20^ ^]^ However, little is known about whether and how IPFP tissue‐derived sEVs (IPFP‐sEVs) exert their roles during OA progression.

Senescence, one of the hallmarks of aging, is a cell fate characterized by irreversible cell cycle arrest and the enhanced secretome of detrimental pro‐inflammatory cytokines into the surrounding microenvironment, which is known as the senescence‐associated secretory phenotype (SASP).^[^ ^21^, ^22^ ^]^ Senescence has been commonly observed in the pathogenesis and progression of degenerative and hyperplastic age‐related diseases.^[^ ^23^, ^24^ ^]^ Senescent cell exhibits increased levels of senescence‐associated beta‐galactosidase (SA‐β‐gal) activity, telomere shortening, and accumulation of nuclear double‐strand DNA damage (increased expressions of p16^INK4a^ and γ‐H2AX).^[^ ^23^, ^25^ ^]^ Recently, numerous reports have indicated the role of chromatin proteins in cellular senescence, such as the nuclear envelope protein, lamin B receptor (LBR).^[^ ^26^ ^]^ Research showed that LBR deficiency promoted excess thymidine‐induced cellular senescence in cancer cells.^[^ ^27^ ^]^ Upregulated expression of LBR suppressed cellular senescence triggered by proteasome inhibitor.^[^ ^26^ ^]^ Evidences were found that inflammatory stimuli, such as IL‐1β, can facilitate senescent changes by enhancing SASP expression in a manner dependent upon oxidative and nitrosative stresses in OA.^[^ ^28^ ^]^ Intra‐articular injection of senescent chondrocytes damages the potential of the mesenchymal stem cells (MSCs) to regenerate cartilage, suggesting that chondrocytes senescence contributes to OA development and progression.^[^ ^29^ ^]^ However, the mechanism of chondrocytes senescence during OA is not fully elucidated.

In this study, we showed that sEVs inhibitor GW4869 efficiently suppressed deleterious effects of IPFP on cartilage. IPFP‐sEVs promoted the cartilage deterioration and chondrocytes senescence in vitro, ex vivo and in vivo. We further demonstrated that let‐7b‐5p and let‐7c‐5p were significantly enriched in IPFP‐sEVs and synovial fluid (SF)‐derived sEVs (SF‐sEVs), which can be transferred to cartilage to regulate cellular senescence by inhibiting LBR. Moreover, Intra‐articular (IA) injection of antagomir‐let‐7b‐5p and let‐7c‐5p could partially alleviate IPFP‐sEVs‐mediated detrimental effects on articular cartilage in vivo. We believe that our findings provide new insights into the role of IPFP‐sEVs and senescence regulators in OA and are valuable for developing new prevention strategies.

## 2 Results

### 2.1 Inhibition of Endogenous sEVs Alleviates Osteoarthritic IPFP‐Induced Cartilage Damages

**Figure 1:**
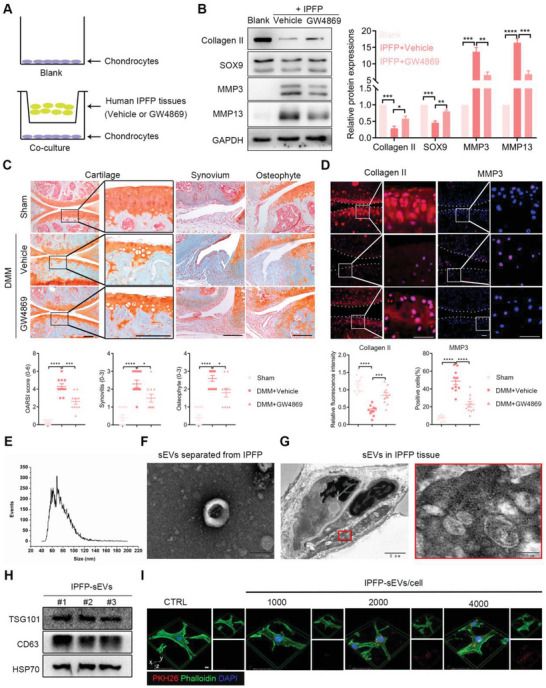
Effects of sEVs inhibitor GW4869 on the roles of IPFP‐sEVs in regulating cartilage metabolism. A) Experimental scheme. B) Transwell assay followed by western blot analysis detecting the expression level of Collagen II, SOX9, MMP3, MMP13 in the co‐cultured chondrocytes. The data was normalized to GAPDH. C) Safranin o/fast green staining of knee joint specimens of mice treated with GW4869 or vehicle (up). Statistical analysis of OARSI, synovitis and osteophyte scores (down). Scale bars, 100 µm. D) Immunofluorescence analysis of Collagen II and MMP3 in sections of knee joints (up, n = 10 for each group). Statistical analysis of immunofluorescence assay (down). Scale bars, 100 µm. E) Size distribution of isolated IPFP‐sEVs. F) Transmission electron microscopy of sEVs isolated from IPFP explant‐conditional medium. Scale bar, 100 nm. G) IPFP tissue consisted of cells and sEVs. Higher magnification pictures showed numerous different types of sEVs in the tissue interstitial space. Scale bars, 2 µm (left), 200 nm (right). H) Indicated proteins CD63, TSG10 and HSP70 were assessed by western blotting from sEVs. I) 3D rendering and Z stack images showing the colocalization of PKH26‐labelled sEVs (red) and phalloidin for F‐actin (green). Scale bar, 5 µm. OARSI, Osteoarthritis Research Society International. ns: no significant difference, *p<0.05, **p<0.01, ***p<0.001, ****p<0.0001. For B) and D), all data are shown as means ± SEM, *p* value were calculated by one‐way analysis of variance (ANOVA). For C), all data are shown as means ± 95% CI, Mann‐Whitney U test was used for OARSI, synovitis and osteophyte scores.

Previous work from our own group have shown that osteoarthritic IPFP exacerbated cartilage degradation and inflammation.^[^ ^30^ ^]^ However, the molecular mechanism of how IPFP influences the degradation of articular cartilage is poorly understood. To assess whether deleterious effects of IPFP on cartilage damages were indeed mediated by sEVs, primary human chondrocytes (HCs) were co‐cultured with osteoarthritic IPFP tissues treated with GW4869 (an inhibitor of sEVs secretion) or vehicle. Consistent with our previous study, HCs co‐cultured with OA IPFP had decreased expressions of Collagen II and SOX9 but increased MMP3 and MMP13. However, this effect was significantly reversed by GW4869 (**Figure** **1**A,B). Next, we further demonstrated the role of endogenous IPFP‐sEVs in experimental OA mouse model induced by destabilization of the medial meniscus (DMM). Safranin O/fast green staining showed that the intra‐articular injection of GW4869 attenuated cartilage damage, synovitis and osteophytes, respectively (Figure 1C). Moreover, the protein expression of Collagen II was increased while MMP3 was decreased in cartilage tissues of GW4869‐treated mice (Figure 1D). These data provide a line of evidences that the pro‐catabolic signal is relayed from IPFP to the cartilage through sEVs.

To directly evaluate the effect of IPFP‐sEVs on articular cartilage, IPFP‐sEVs were isolated from IPFP tissue conditioned medium and purified by ultracentrifugation (Figure S1, Supporting Information). Similar to previously described sEVs,^[^ ^17^ ^]^ examination of the size distribution of IPFP‐sEVs by Nanoparticle Tracking Analysis (NTA) revealed that the majority of the population was in the 30–200 nm range (Figure 1E). Moreover, the purified IPFP‐sEVs were displayed an ovoid morphology, as shown by transmission electron microscopy (TEM) (Figure 1F). We also found that the existence of sEVs in the interstitial space of IPFP tissue by electron microscopy (Figure 1G). Moreover, we further confirmed that the sEVs positive markers TSG101, CD63 and HSP70 were abundant in the sEVs‐derived fractions (Figure 1H). sEVs are important mediators of intercellular and interorgan communication, which are released from tissues or cells and then absorbed by target cells to exert biological functions.^[^ ^31^ ^]^ To explore whether IPFP‐sEVs could be taken up by chondrocytes, HCs were co‐cultured with IPFP‐sEVs for 24 h. Red fluorescence‐marked IPFP‐sEVs were located in the green fluorescence‐labelled HCs cytoskeleton, confirming that IPFP‐sEVs were internalized successfully (Figure 1I). Together, these data suggest that osteoarthritic IPFP tissues secrete sEVs, which could be taken by chondrocytes.

### 2.2 OA IPFP‐sEVs Promote Cartilage ECM Degradation and Chondrocytes Senescence

**Figure 2:**
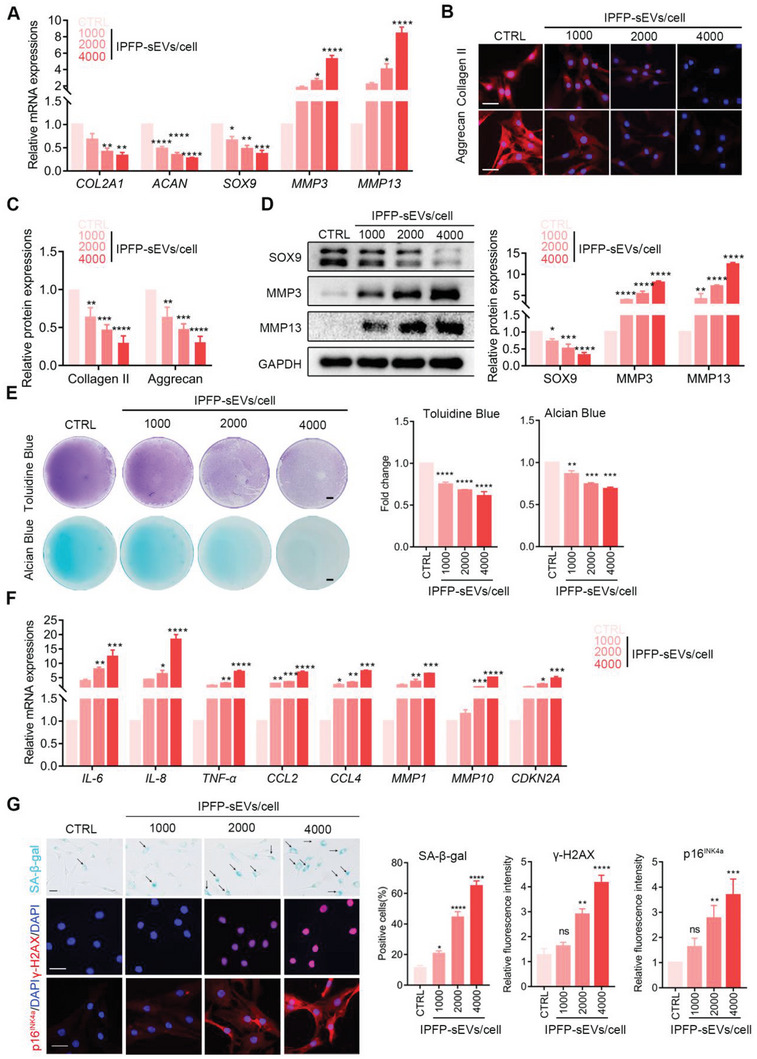
IPFP‐sEVs promote ECM degradation and cellular senescence in HCs. A) qRT‐PCR analysis of *COL2A1*, *ACAN*, *SOX9*, *MMP3* and *MMP13* expression in HCs stimulated with IPFP‐sEVs at 0 (control), 1000, 2000, or 4000 IPFP‐sEVs cell^−1^ for 24 h. B) Representative immunofluorescence images of of Collagen II and Aggrecan protein levels in chondrocytes after IPFP‐sEVs stimulation. Scale bars, 25 µm. C) Quantification data of Fig 2B. D) Western blotting analysis of SOX9, MMP3 and MMP13 expression in chondrocytes after stimulating with IPFP‐sEVs. E) Alcian Blue and Toluidine Blue staining after a 14‐day treatment with IPFP‐sEVs or PBS. Scale bars, 5 µm. F) qRT‐PCR analysis of *IL‐6*, *IL‐8*, *TNF‐α*, *CCL2*, *CCL4*, *MMP1*, *MMP10* and *CDKN2A* expression in HCs stimulated with IPFP‐sEVs at 0 (control), 1000, 2000, or 4000 IPFP‐sEVs cell^−1^. G) SA‐β‐gal staining (left) representing the effects of IPFP‐sEVs on senescence and SA‐β‐gal positive cell counting (right) of HCs. Scale bar, 100 µm. Representative immunofluorescence images and quantification fluorescence intensity analyses of γ‐H2AX and p16^INK4a^ protein levels in chondrocytes after IPFP‐sEVs stimulation. Scale bars, 25 µm. ns: no significant difference, *p<0.05, **p<0.01, ***p<0.001, ****p<0.0001. All data are shown as means ± SEM of three independent experiments in A), C), D), E), F) and G). One‐way ANOVA were used for comparison between multiple groups.

Next, to explore the roles of IPFP‐sEVs in chondrocytes metabolism, IPFP‐sEVs were used to stimulate HCs at a concentration gradient. The results of qRT‐PCR assays showed that the IPFP‐sEVs significantly inhibited *COL2A1, ACAN* and *SOX9* mRNA levels and increased *MMP3* and *MMP13* mRNA expressions (**Figure** **2**A). Consistent with the mRNA levels, the protein levels of Collagen II, Aggrecan and SOX9 in chondrocytes were significantly decreased, while the expressions of MMP3 and MMP13 were enhanced with the increase of IPFP‐sEVs concentration (Figure 2B–D). Furthermore, IPFP‐sEVs‐induced ECM degradation in HCs was confirmed by alcian blue and toluidine blue staining (Figure 2E).

Previous studies have demonstrated that senescence plays an deteriorate role in cartilage homeostasis.^[^ ^23^ ^]^ Senescent cells could secrete a complex mixture of soluble factors with proinflammatory, ECM remodeling and immunomodulatory activities.^[^ ^21^ ^]^ To investigate whether IPFP‐sEVs induce senescence in HCs, we performed qRT‐PCR assay to detect the expressions of SASP, such as *IL‐6*, *IL‐8*, *TNF‐α*, *CCL2*, *MMP1*, *MMP10*, *CDKN2A* and *CCL4*. These genes were increased markedly in HCs with IPFP‐sEVs stimulation (Figure 2F). Furthermore, SA‐β‐gal staining revealed that IPFP‐sEVs significantly increased the number of senescent chondrocytes in a concentration‐dependent manner (Figure 2G), while immunofluorescence of γ‐H2AX and p16^INK4a^ were further conducted to demonstrate the effect of IPFP‐sEVs on HCs senescence (Figure 2G). In order to identify the effect of sEVs from normal IPFP, we collected three normal IPFP tissue samples and isolated their sEVs for further experiments. Normal IPFP‐sEVs were used to stimulate HCs at a concentration gradient. ECM metabolism and cellular senescence were evaluated by western blotting and SA‐β‐gal staining, respectively. Interestingly, the results showed that normal IPFP‐sEVs did not induce ECM degradation and chondrocytes senescence at the same concentration gradient as OA IPFP‐sEVs did (Figure S2A,B, Supporting Information). These results suggest that OA IPFP‐sEVs, rather than normal IPFP‐sEVs, coud not only aggravate ECM degradation but also promote cellular senescence in HCs.

### 2.3 IPFP‐sEVs Accelerate OA Development and Progression Ex Vivo and In Vivo

**Figure 3:**
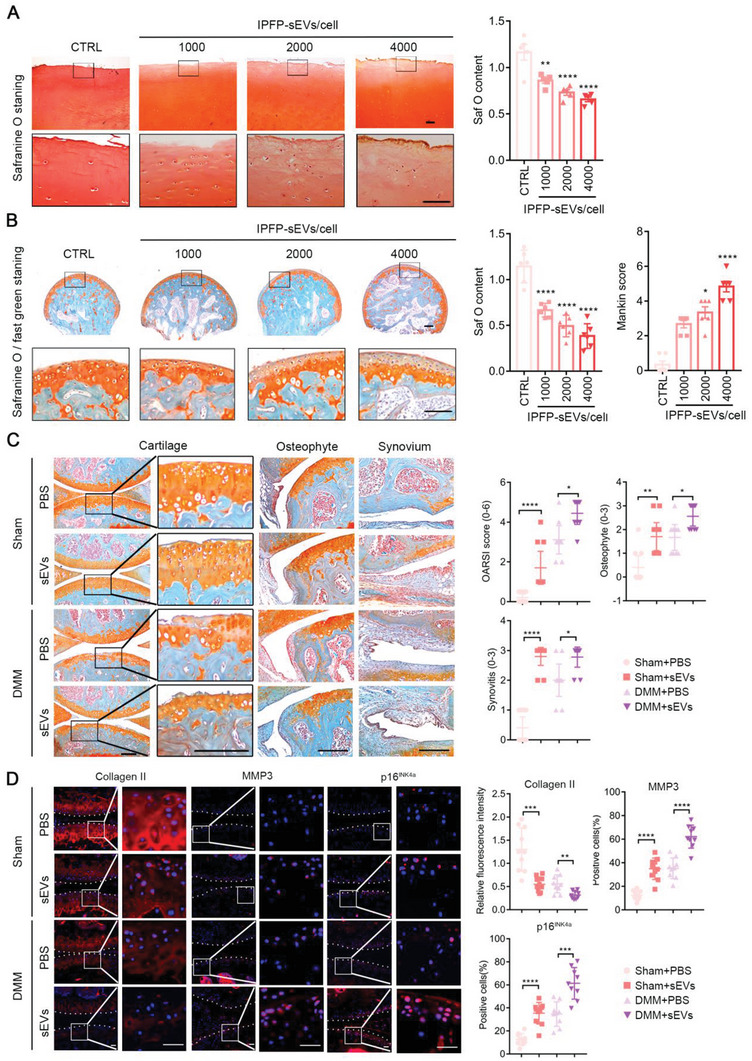
IPFP‐sEVs accelerate OA development and progression *ex vivo* and in vivo. A) Representative safranin O‐stained images of human cartilage explants treated with or without IPFP‐sEVs. Scale bars, 25 µm. Safranin O‐positive area among the above groups was quantified (*n* = 5). B) Mice femoral head treated with or without IPFP‐sEVs. The cartilage was stained with safranin‐o/fast green. Scale bars, 25 µm. The OA severity was accessed by relative safranin‐o staining content and Mankin score (*n* = 8). C) Mice in sham and DMM‐induced OA group were injected with IPFP‐sEVs or PBS, separately. The cartilage, osteophyte and synovium were stained with safranin‐o/fast green. Scale bars, 100 µm. (*n* = 10 mice in sham group, n = 9 in OA group). OARSI score for articular cartilage. Synovium score for synovitis. Osteophyte score for osteophyte formation. D) Representative immunofluorescence analysis of Collagen II, MMP3 and p16^INK4a^ in sections of both femoral head and tibial plateau of knee joints (*n* = 10 for sham group, *n* = 9 for OA group). Scale bar: 100 µm. ns: no significant difference, *p<0.05, **p<0.01, ***p<0.001, ****p<0.0001. For analysis of Saf O content in A) and B), all data are shown as means ± SEM, one‐way ANOVA were used for comparison between multiple groups. For B) of Mankin score and C), all data are shown as means ± 95% CI, Mann‐Whitney U test was applied for Mankin, OARSI, synovitis and osteophyte scores. For D), Student's *t* test was used for comparison between two groups.

To examine the effects of OA IPFP‐sEVs on cartilage tissue, human cartilage explants were incubated with or without IPFP‐sEVs ex vivo for 14 days. Loss of proteoglycans were observed through safranin o staining in IPFP‐sEVs‐treated human cartilage explants (**Figure** **3**A). We further harvested femoral head cartilage explants from 4‐month‐old wild‐type mice and then stimulated them with IPFP‐sEVs for 14 days. To verify whether IPFP‐sEVs can penetrate and infiltrate into cartilage tissue, mice cartilage explants were incubated with PKH26‐labeled IPFP‐sEVs for 24 or 48 h. Fluorescent signal appeared on cartilage surface at 24 h, while a strong fluorescent signal was present in the deep calcified layer at 48 h (Figure S3, Supporting Information), indicating that IPFP‐sEVs were able to penetrate into mice cartilage tissue. Subsequently, proteoglycans loss was observed remarkably in IPFP‐sEVs‐stimulated mice cartilage explants (Figure 3B).

To further demonstrate the role of IPFP‐sEVs in OA development and progression, we explored the biological effect of IPFP‐sEVs in vivo. Intra‐articular (IA) injection was conducted in sham mice and DMM‐induced OA mice with IPFP‐sEVs or PBS for 10 weeks (Figure S4A, Supporting Information). Safranin o/fast green staining showed that IPFP‐sEVs in both groups could aggravate cartilage destruction and the formation of synovitis and osteophyte (Figure 3C; Figure S4B, Supporting Information). To analyse the effect of IPFP‐sEVs on the catabolic activities of cartilage, pathological alterations in articular cartilage were examined. The injection of IPFP‐sEVs aggravated the deteriorative process in the cartilage matrix as showed decreased anabolic response (Collagen II), increased catabolic response (MMP3) and senescent cells (p16^INK4a^) by immunofluorescence (Figure 3D). All together, these results clearly revealed that IPFP‐sEVs could accelerate OA development and progression ex vivo and in vivo.

### 2.4 Let‐7b‐5p and let‐7c‐5p are Highly Enriched in OA IPFP‐sEVs and Could be Transferred into Cartilage

**Figure 4:**
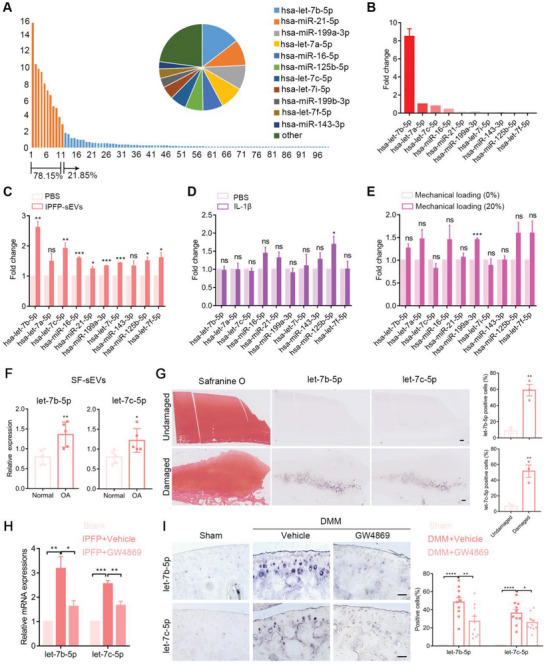
Highly expressed let‐7b‐5p and let‐7c‐5p in IPFP‐sEVs could be transferred from synovial fluid to cartilage tissues. A) Relative proportion of miRNAs in total miRNA reads. Top 10 sEVs‐derived miRNAs expression assessed by miRNA microarray analysis. B) High expression of top 10 miRNAs in IPFP‐sEVs were confirmed by qRT‐PCR. C) qRT‐PCR analysis of top 10 miRNAs expression in HCs stimulated with IPFP‐sEVs for 24 h. D) qRT‐PCR analysis of top 10 miRNAs expression in HCs stimulated with IL‐1β for 24 h. E) qRT‐PCR analysis of top 10 miRNAs expression in HCs after excessive mechanical loading (20%) for 24 h. F) qRT‐PCR analysis of the expression of let‐7b‐5p and let‐7c‐5p in synovial fluid (SF)‐derived sEVs (SF‐sEVs). G) Safranin O staining (left) and ISH (right) of the cartilage from OA patients. Scale bars, 5 µm. H) qRT‐PCR analysis of the expression of let‐7b‐5p and let‐7c‐5p in the co‐cultured chondrocytes. I) ISH of cartilage from sham and DMM mice treated with or without GW4869. Scale bars, 100 µm. ns: no significant difference, *p<0.05, **p<0.01, ***p<0.001, ****p<0.0001. All data are shown as means ± SEM in C), D), E), F), G), H) and I). Student's *t* test was used for comparison between two groups for C), D), E), F) and G). One‐way ANOVA were used for comparison between multiple groups for H) and I).

Among the sEVs cargos, miRNAs have been studied most completely and are highly conserved among species.^[^ ^17^ ^]^ Therefore, we sought to uncover the miRNA expression profiles of sEVs derived from IPFP tissue by miRNA sequencing. The data analysis suggested that the top 10 abundant miRNAs accounted for 78.15% of the total miRNA reads and were listed in pie chart (**Figure** **4**A). qRT‐PCR analysis further confirmed the expression pattern of these miRNAs and suggested that let‐7b‐5p, let‐7a‐5p and let‐7c‐5p were the top 3 miRNAs in IPFP‐sEVs (Figure 4B). To narrow down the candidates, we detected these miRNAs in IPFP‐sEVs‐treated HCs. The results showed that let‐7b‐5p and let‐7c‐5p were the most two differentially expressed miRNAs in IPFP‐sEVs‐treated HCs (Figure 4C). Interestingly, the expression of let‐7b‐5p and let‐7c‐5p showed no significant differences in IL‐1β‐ or excessive mechanical loading‐treated HCs (Figure 4D,E). These results suggested that the increased expression of let‐7b‐5p and let‐7c‐5p in chondrocytes were predominantly due to IPFP‐sEVs rather than inflammatory factor or mechanical overloading. More importantly, we also found the increased expression of let‐7b‐5p and let‐7c‐5p in human osteoarthritic SF‐sEVs by qRT‐PCR and OA articular cartilage by in situ hybridization (ISH) staining (Figure 4F,G). Moreover, the expression of let‐7b‐5p and let‐7c‐5p could be partially downregulated in HCs by inhibiting IPFP‐sEVs production in vitro (Figure 4H). We also found that inhibition of IPFP‐sEVs decreased the expression of let‐7b‐5p and let‐7c‐5p in articular cartilage in mice OA model (Figure 4I). To determine the origin of sEVs, we performed ISH staining of let‐7b‐5p and let‐7c‐5p in IPFP samples. The results showed let‐7b‐5p and let‐7c‐5p in OA IPFP tissues were mostly expressed in blood vessel (Figure S5, Supporting Information). Collectively, these results indicated that let‐7b‐5p and let‐7c‐5p were enriched in IPFP‐sEVs and could be transferred into cartilage.

### 2.5 Let‐7b‐5p and let‐7c‐5p are Essential for IPFP‐sEVs‐Mediated Effects on Chondrocytes Degradation and Senescence

**Figure 5:**
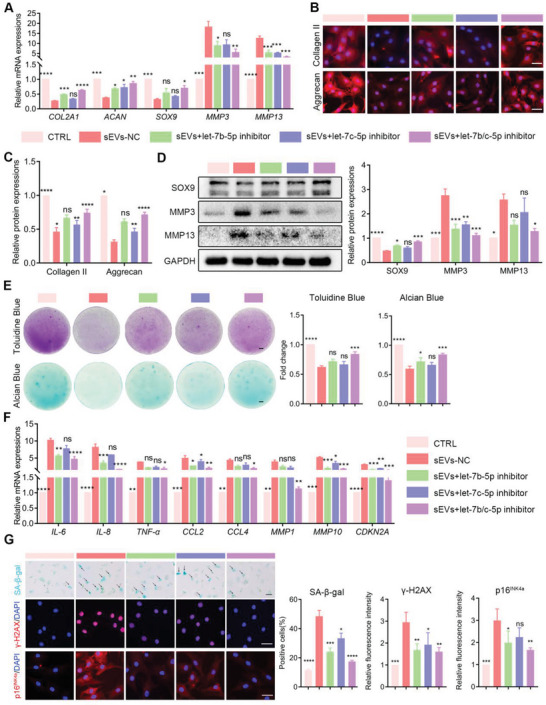
Inhibition of let‐7b‐5p and let‐7c‐5p suppresses biological functions of IPFP‐sEVs in chondrocytes. A) qRT‐PCR analysis of *COL2A1*, *ACAN*, *SOX9*, *MMP3* and *MMP13* expression in HCs after IPFP‐sEVs stimulation or a combination of IPFP‐sEVs and let‐7b‐5p or let‐7c‐5p inhibition. B) Representative immunofluorescence images of Collagen II and Aggrecan after IPFP‐sEVs treatment or/with let‐7b‐5p and let‐7c‐5p inhibition. C) Quantification data of Figure 5B. D) Western blotting analysis of SOX9, MMP3 and MMP13 protein levels in chondrocytes after IPFP stimulation or a combination of IPFP‐sEVs and let‐7b‐5p or let‐7c‐5p inhibition. The data were normalized to GAPDH. E) Alcian Blue and Toluidine Blue staining were performed to examine the proteoglycan loss of HCs treated with the IPFP‐sEVs with or without let‐7b‐5p and let‐7c‐5p inhibitor (left) and its quantification data (right). Scale bar: 5 µm. F) qRT‐PCR analysis of *IL‐6*, *IL‐8*, *TNF‐α*, *CCL2*, *CCL4*, *MMP1*, *MMP10* and *CDKN2A* expression in HCs as treated above. G) Identification of senescent cells by SA‐β‐gal staining as treated above (left) and its quantification data (right). scale bar: 100 µm. Immunofluorescence staining of p16^INK4a^ and γ‐H2AX in chondrocytes as treated above (left) and its quantification data (right). Scale bars: 25 µm. ns: no significant difference, *p<0.05, **p<0.01, ***p<0.001, ****p<0.0001. All data are shown as means ± SEM of three independent experiments in A), C), D), E), F) and G). One‐way ANOVA were used for comparison in multiple groups.

Next, we test whether the effect of IPFP‐sEVs on ECM metabolism and chondrocytes senescence were implemented through let‐7b‐5p and let‐7c‐5p. Indeed, transfection of let‐7b‐5p or let‐7c‐5p inhibitor into chondrocytes reversed the effect of IPFP‐sEVs on downregulating the expressions of *COL2A1, ACNA* and *SOX9* mRNAs and increasing the expressions of *MMP3* and *MMP13* mRNAs (**Figure** **5**A). Changes in the protein levels of these ECM anabolic and catabolic markers also endorsed this reversed effect by western blotting analysis and immunofluorescent staining (Figure 5B–D). Furthermore, increased proteoglycan loss was observed in the IPFP‐sEVs‐stimulated HCs, while this effect could be blocked by let‐7b‐5p and let‐7c‐5p inhibition (Figure 5E).

Notably, in vitro studies attempted to determine whether let‐7b‐5p and let‐7c‐5p silence led to senescence inhibition. The mRNA expression levels of SASP were significantly increased in IPFP‐sEVs‐treated HCs, whereas in the let‐7b‐5p and let‐7c‐5p inhibitor group, these mRNAs were downregulated (Figure 5F). SA‐β‐gal staining showed that the increased senescent HCs induced by IPFP‐sEVs were reversed by let‐7b‐5p and let‐7c‐5p inhibitor (Figure 5G). Moreover, immunofluorescence assays of γ‐H2AX and p16^INK4a^ further confirmed the roles of let‐7b‐5p and let‐7c‐5p (Figure 5G). Collectively, our data demonstrated that IPFP‐sEVs promoted the ECM catabolism and chondrocytes senescence by delivering let‐7b‐5p and let‐7c‐5p.

### 2.6 IPFP‐sEVs‐Packaged let‐7b‐5p and let‐7c‐5p Exert Biological Functions by Targeting LBR

**Figure 6:**
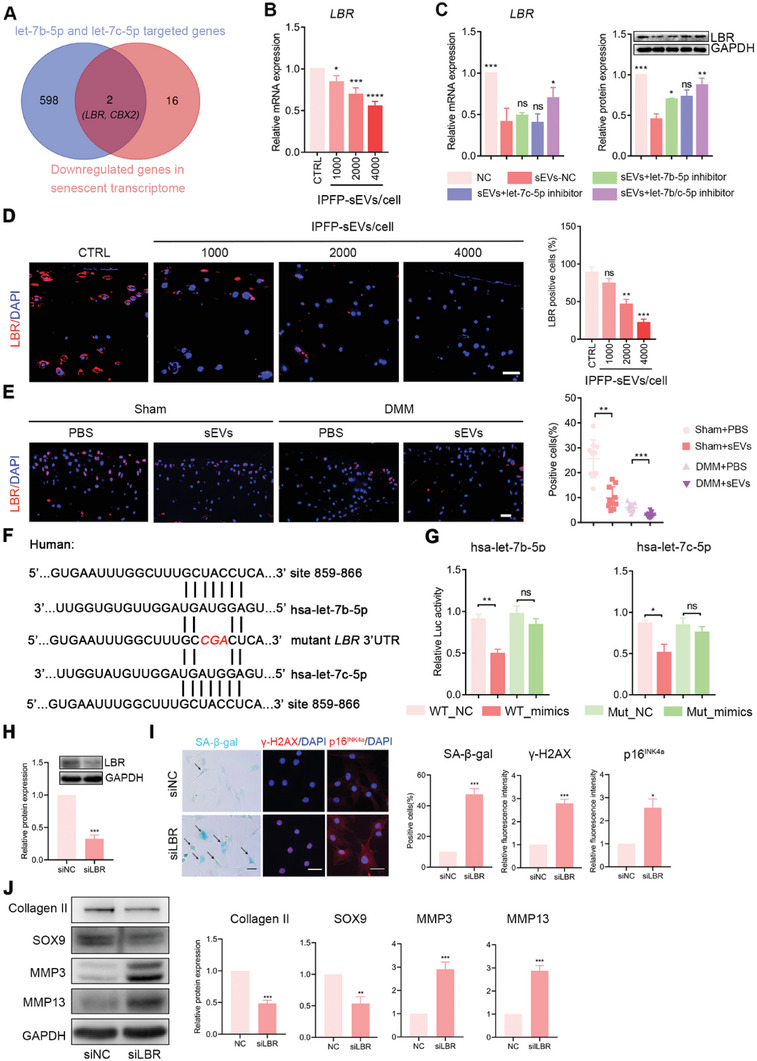
Let‐7b‐5p and let‐7c‐5p directly target LBR. A) Venn diagram display the overlapping of the human target genes of let‐7b‐5p and let‐7c‐5p, as predicted by Targetscan, miRDB, miRPathDB, starbase and senescent core genes. B) qRT‐PCR analysis of *LBR* in HCs stimulated with IPFP‐sEVs for 0 (control), 1000, 2000, or 4000 IPFP‐sEVs cell^−1^ for 24 h. C) mRNA expression of *LBR* was measured by qRT‐PCR in chondrocytes after IPFP stimulation or a combination of IPFP‐sEVs and let‐7b/c‐5p inhibition (left). Western blotting analysis of LBR protein level in chondrocytes as treated above (right). The data was normalized to GAPDH. D) Representative images of LBR assayed by immunofluorescence confocal microscopy in IPFP‐sEVs‐stimulated human cartilage explants. Scale bar, 25 µm. E) Representative immunofluorescence staining of LBR in mice model. Scale bar, 25 µm. F) Sequence alignment of a putative let‐7b‐5p and let‐7c‐5p binding site within the 3′‐UTR of *LBR* mRNA shows a high level of sequence conservation and complementarity with let‐7b‐5p and let‐7c‐5p. G) Luciferase assays show decreased reporter activity after co‐transfection of the wild‐type LBR 3′UTR plasmid with let‐7b‐5p and let‐7c‐5p into 293T cells. H) The knockout efficiency was detected by western blotting analysis. I) Identification of senescent cells by SA‐β‐gal staining as treated above (left) and its quantification data (right). Scale bar: 100 µm. Representative immunofluorescence images and quantification fluorescence intensity analysis of γ‐H2AX and p16^INK4a^ protein levels in chondrocytes after transfection of siLBR. Scale bars, 25 µm. J) Western blotting analysis of Collagen II, SOX9, MMP3 and MMP13 expression in chondrocytes after transfection of siLBR. ns: no significant difference, *p<0.05, **p<0.01, ***p<0.001, ****p<0.0001. All data are shown as means ± SEM of three independent experiments in B), C), D), G), H), I) and J). One‐way ANOVA was used for comparison in multiple groups for B), C) and D). Student's *t* test was used in E), G), H), I) and J).

To uncover the molecular mechanisms underlying let‐7b‐5p and let‐7c‐5p‐regulated chondrocyte homeostasis, we performed bioinformatics analysis with human public database, including miRDB, TargetScan, starbase and miRPathDB and merged them with 18 downregulated core genes in senescent transcriptome.^[^ ^32^ ^]^ The result showed that *LBR* and *CBX2* could be the potential targeted genes of let‐7b‐5p and let‐7c‐5p (**Figure** **6**A). qRT‐PCR analysis demonstrated that both *LBR* and *CBX2* mRNA expression levels were downregulated in IPFP‐sEVs‐treated HCs (Figure 6B; Figure S6A, Supporting Information). Next, we performed rescue assays to further confirm the downstream target of let‐7b‐5p and let‐7c‐5p. qRT‐PCR and western blotting analysis suggested that the *LBR* mRNA and protein (LBR) levels were negatively linked to let‐7b‐5p and let‐7c‐5p in IPFP‐sEVs‐treated HCs, while CBX2 expression did not show significant difference (Figure 6C; Figure S6B,C, Supporting Information). Furthermore, immunofluorescence analysis revealed that LBR expression was significantly decreased in the human OA‐damaged cartilage (Figure S7A, Supporting Information). Similar results were also identified in the experimental mice OA model (Figure S7B, Supporting Information). We further found lower LBR expression levels in IPFP‐sEVs‐treated HCs, whereas inhibition of let‐7b‐5p and let‐7c‐5p significantly reversed this change (Figure S7C, Supporting Information). We also assessed the expression of LBR in human cartilage explants and experimental mice OA model. Consistently, LBR expression was decreased in IPFP‐sEVs stimulated human cartilage explants in a dose‐dependent manner (Figure 6D). Moreover, the expression level of LBR was also downregulated in articular cartilage from sham and DMM mice after IA injection of IPFP‐sEVs (Figure 6E). Furthermore, target prediction algorithms illustrated that human and mouse *LBR* 3′UTR regions were defined as the target of let‐7b‐5p and let‐7c‐5p, which contained putative seed sequences (Figure 6F; Figure S7D, Supporting Information). To further verify whether let‐7b‐5p and let‐7c‐5p directly binds with the 3′‐UTR of *LBR* mRNA, we proceeded luciferase reporter assay. Transfection with let‐7b‐5p and let‐7c‐5p mimics resulted in reduced luciferase activity by binding to the wild‐type 3′‐UTR of *LBR*, while the mutant 3′‐UTR sequence blocked the binding of let‐7b‐5p and let‐7c‐5p (Figure 6G). To further clarify the relationship between LBR‐related senescence and cartilage degradation, we performed LBR inhibition by siRNA in HCs. Western blotting results showed a significant decrease in LBR expression level after siRNA treatment (Figure 6H). LBR knockdown in chondrocytes increased the cellular senescence (Figure 6I) and ECM catabolic proteins (such as MMP3 and MMP13), but decreased anabolic proteins (such as Collagen II and SOX9) (Figure 6J). Therefore, our results demonstrated that LBR inhibition‐induced chondrocytes senescence could contribute to chondrocyte degradation. Taken together, our data show that LBR expression is decreased in OA cartilage and IPFP‐sEVs‐delivered let‐7b‐5p and let‐7c‐5p exert biological functions by targeting LBR.

### 2.7 Inhibition of let‐7b‐5p and let‐7c‐5p Alleviates IPFP‐sEVs‐Induced OA Progression in Mice

**Figure 7:**
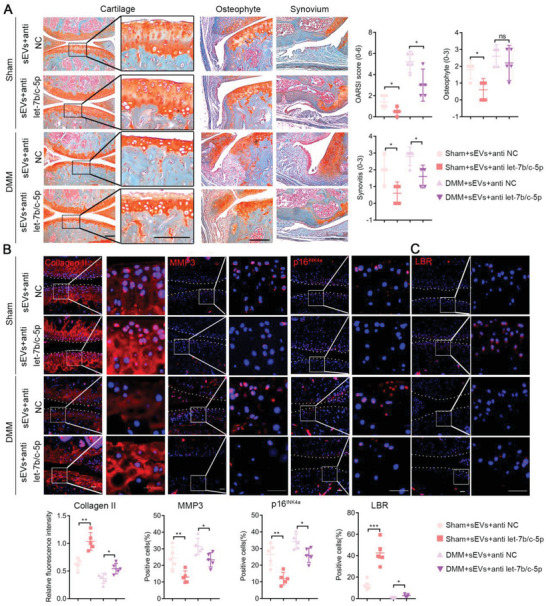
Intra‐articular injection of antagomir‐let‐7b‐5p and let‐7c‐5p demolishes the deteriorative effect of IPFP‐sEVs on knee joint in vivo. A) Safranin o/fast green staining of knee joint specimens of mice treated with antagomir‐NC or antagomir‐let‐7b‐5p and let‐7c‐5p (left). Statistical analysis of OARSI, synovitis and osteophyte scores (right). Scale bar: 100 µm. B,C) Representative immunofluorescence analysis of Collagen II, MMP3, p16^INK4a^ B) and LBR C) in sections of both femoral head and tibial plateau of knee joints (*n* = 5 for each group). Scale bar: 25 µm. ns: no significant difference, *p<0.05, **p<0.01, ***p<0.001, ****p<0.0001. For A), all data are shown as means ± 95% CI, Mann‐Whitney U test for OARSI, synovitis and osteophyte scores. For B) and C), all data are shown as means ± SEM, Student's *t* test was used for comparison.

Considering the key role of anti‐let‐7b‐5p and let‐7c‐5p in ECM homeostasis in vitro, we sought to test whether silence of let‐7b‐5p and let‐7c‐5p had OA therapeutic effects in vivo (Figure S8A, Supporting Information). Cartilage, synovium and osteophyte tissue sections of the knee joint were stained with safranin o/fast green and then morphologically observed. The cartilage damage in the mice treated with antagomir‐let‐7b‐5p and let‐7c‐5p was significantly reduced and the structure of the cartilage surface was restored, suggesting that impeded let‐7b‐5p and let‐7c‐5p contained in IPFP‐derived sEVs prevented cartilage damage (**Figure** **7**A). Furthermore, let‐7b‐5p and let‐7c‐5p‐inhibited knees exhibited a significantly thin synovial lining in the synovitis scores and reduced osteophytes, especially in sham groups (Figure 7A). We further characterized the late OA symptom of osteophyte formation by micro‐CT. The results demonstrated that osteophytes were frequently found in control‐treated groups but not in antagomir‐let‐7b‐5p and let‐7c‐5p‐treated group (Figure S8B, Supporting Information). To gain further insight into the impact of antagomir‐let‐7b‐5p and let‐7c‐5p‐sEVs on cartilage degradation and senescence in vivo, we detected the expression of matrix metabolic enzymes and senescence genes in the knee joints of both sham and DMM‐induced OA mice by using immunofluorescence analysis. The results demonstrated that IPFP‐sEVs lowered the level of Collagen II and increased the expression of MMP3 and p16^INK4a^, while the antagomir‐let‐7b‐5p and let‐7c‐5p impeded this effect of IPFP‐sEVs on the indicated protein levels in vivo (Figure 7B). We also found that the lower expression of LBR after IPFP‐sEVs stimulation could be significantly reversed by let‐7b‐5p and let‐7c‐5p inhibition (Figure 7C). Taken together, these data demonstrate that inhibiting the expression of let‐7b‐5p and let‐7c‐5p in IPFP‐sEVs may be an effective therapy for cartilage protection in OA progression.

## 3 Discussion

To our knowledge, this is the first study to examine the effect of IPFP tissue‐derived sEVs on cartilage metabolism and cellular senescence in OA. Inhibition of endogenous osteoarthritic IPFP‐sEVs significantly alleviated cartilage damages. We demonstrated that IPFP‐sEVs significantly promoted chondrocyte degradation and cell aging by delivering let‐7b‐5p and let‐7c‐5p, which inhibited senescence negative regulator LBR. Notably, IA injection of antagomirs inhibiting let‐7b‐5p and let‐7c‐5p in mice increased LBR expression, suppressed chondrocyte senescence and ameliorated the progression of experimental OA model.

IPFP, an integral part of the knee joint, is capable of generating and secreting inflammatory mediators directly within the joint, thereby affecting the evolution and progression of OA.^[^ ^9^ ^]^ Previous studies by our group have revealed that osteoarthritic IPFP augments the inflammation and degradation of cartilage.^[^ ^30^ ^]^ In agreement with the study, we found that IPFP from end‐stage OA could induce cartilage degradation by co‐culturing with HCs, but this effect can be reversed after GW4869 administration. We further confirmed that IPFP‐sEVs exerted detrimental effects by promoting cartilage degradation, implying that IPFP act in OA progression may be due to the role of sEVs in IPFP tissues. Other studies noted that aberrant IPFP could produce a variety of pro‐inflammatory cytokines such as IL‐1β, TNF‐α, IL‐6 and IL‐8, as well as adipokines such as leptin and resistin, and therefore might play a deleterious role in OA, but no studies have further demonstrated a direct effect of IPFP on senescence.^[^ ^8^ ^]^ This study was the first to verify that IPFP‐sEVs‐stimulated chondrocytes could increase the expression of SASP and senescence‐related DNA damage, which further confirm the importance of IPFP‐sEVs in OA‐induced pathology.

Using an *ex vivo* IPFP tissue culture system, we provided evidence that sEVs could be secreted from IPFP tissues. Unlike the data for the sEVs released from in vitro cultured cells, this study showed that the data generated using sEVs released from IPFP tissues are more relevant to what may occur in vivo. To our knowledge, this study highlights the biological significance of IPFP‐sEVs in the progression of OA. Of note, while IPFP‐sEVs are first found in the tissue interstitial space and may be most likely to act locally, IPFP‐sEVs carrying let‐7b‐5p and let‐7c‐5p can be released into the knee joint. Therefore, synovial liquid biopsy is a promising alternative diagnostic approach, and biomarkers might be found in IPFP‐sEVs‐based mechanism researches.

Most sEVs functions have been attributed to miRNAs that act on target mRNAs to trigger their degradation or prevention of translation.^[^ ^33^ ^]^ Importantly, miRNAs in sEVs could be released into circulation, and mediate inter‐organ communication.^[^ ^34^ ^]^ Over the past few decades, sEVs‐derived miRNAs have been recognized as critical regulators in the pathophysiological process of OA.^[^ ^35^, ^36^ ^]^ Here, we demonstrated that as an especially important contributor to the pool of circulating miRNAs, let‐7b‐5p and let‐7c‐5p in IPFP‐sEVs could be transferred to synovial fluid and then induce chondrocyte degradation and senescence in OA. Previous studies have indicated that let‐7b‐5p in adipose tissue‐derived stem cells EVs inhibited osteoclast differentiation and significantly reduced the expression of genes related to bone resorption.^[^ ^37^ ^]^ In addition, highly expressed let‐7b‐5p in sEVs induced apoptosis and repressed proliferation migration and matrix synthesis of annulus fibrosus cells.^[^ ^38^ ^]^ Let‐7c‐5p, the family of let‐7b‐5p, negatively regulates expression levels of IL‐1β, IL‐6 and TNF‐α, pinpointing a novel strategy for reducing OA inflammation.^[^ ^39^ ^]^ Moreover, let‐7c‐5p regulates cell proliferation and apoptosis in many other diseases.^[^ ^40^, ^41^ ^]^ In our study, we found that let‐7b‐5p and let‐7c‐5p were highly expressed in osteoarthritic IPFP‐sEVs and SF‐sEVs. Knockdown of let‐7b‐5p and let‐7c‐5p significantly reversed the pro‐senescence effect and cartilage destruction, confirming that IPFP‐sEVs‐derived let‐7b‐5p and let‐7c‐5p could be delivered into cartilage to accelerate ECM degeneration and chondrocytes senescence. Hence, we hypothesized that maintaining let‐7b‐5p and let‐7c‐5p at the physiological level may be critical for cartilage homeostasis. Interestingly, we found that let‐7b‐5p and let‐7c‐5p in OA IPFP tissues were mostly expressed in blood vessel, suggesting that vessel formation in OA IPFP could be the key harmful factor. Blood vessels in OA IPFP could be closely related to the development of tissue inflammation, fibrosis, and pain‐related nerve fiber formation.^[^ ^42^, ^43^, ^44^ ^]^ Our study suggests that blood vessels in OA IPFP may be the main source of its harmful sEVs, indicating that further investigation into blood vessels is of great significance.

LBR is an integral membrane protein embedded in the inner nuclear envelope with structural impact on nuclear shape and chromatin organization.^[^ ^26^, ^27^ ^]^ LBR is involved in cellular senescence process, and its expression is decreased in senescent cells.^[^ ^45^ ^]^ Several studies have showed that miRNAs affect cartilage senescence by suppressing or initiating LBR expression, thus relieving or exacerbating diseases progression.^[^ ^45^, ^46^ ^]^ For example, LBR was identified as a direct miR‐222 target. MiR‐222 overexpression, or LBR knockdown in breast normal fibroblasts induced cell senescence.^[^ ^46^ ^]^ Furthermore, miR‐340‐5p promoted senescence was through its direct impact on *LBR* mRNA.^[^ ^45^ ^]^ In our study, we analyzed the downstream target genes of let‐7b‐5p and let‐7c‐5p and identified that let‐7b‐5p and let‐7c‐5p may target various genes. However, our results indicated that let‐7b‐5p and let‐7c‐5p were related to senescence factors, and could directly target LBR, which could deter chondrocytes senescence. Moreover, LBR was downregulated in the cartilage damaged zone, and regulated by IPFP‐sEVs‐derived let‐7b‐5p and let‐7c‐5p. Hence, these findings point out potential molecular targets and mechanism for senescence induction and therapeutic strategies in OA.

This study has limitations. First, due to the difficulty in collecting normal IPFP, the first limitation of this study is whether the function and mechanism of IPFP‐sEVs from OA patients are different from that of normal IPFP. Second, IPFP tissue is mostly originated from end‐stage knee OA patient who have already opted for surgical treatment, so whether IPFP‐sEVs could be used for early disease diagnosis warrants further investigation. Third, it is noteworthy that although GW4869 is widely used as sEVs inhibitor, in vivo GW4869 treatment may cause several nonspecific effects. Last, how let‐7b‐5p and let‐7c‐5p are packaged into the sEVs remains unclear. Further investigation will be carried out to explore these limitations in future.

In summary, our findings suggest that sEVs in IPFP from OA patients impair cartilage metabolism and induce chondrocyte senescence. This study will add to the growing understanding of the role of IPFP‐sEVs and would provide a new therapeutic target for OA.

## Conflict of Interest

The authors declare no conflict of interest.

## Supporting information

Supporting Information

## Data Availability

The data that support the findings of this study are available in the supplementary material of this article.

## References

[1] D. J. Hunter , S. Bierma‐Zeinstra , Lancet 2019, 393, 1745. 31034380 10.1016/S0140-6736(19)30417-9

[2] A. Ratneswaran , M. Kapoor , Osteoarthritis cartilage 2021, 29, 151. 33227439 10.1016/j.joca.2020.11.003

[3] J. G. Quicke , P. G. Conaghan , N. Corp , G. Peat , Osteoarthritis cartilage 2022, 30, 196. 34695571 10.1016/j.joca.2021.10.003

[4] S. J. Rice , F. Beier , D. A. Young , J. Loughlin , Nat Rev. Rheumatol 2020, 16, 268. 32273577 10.1038/s41584-020-0407-3

[5] N. Zeng , Z.‐P. Yan , X.‐Y. Chen , G.‐X. Ni , Aging Dis 2020, 11, 1317. 33014539 10.14336/AD.2019.1116 PMC7505265

[6] W. Han , S. Cai , Z. Liu , X. Jin , X. Wang , B. Antony , Y. Cao , D. Aitken , F. Cicuttini , G. Jones , C. Ding , Arthritis Res. Ther. 2014, 16, R145. 25008048 10.1186/ar4607 PMC4227074

[7] M. Richter , T. Trzeciak , M. Owecki , A. Pucher , J. Kaczmarczyk , Int Orthop 2015, 39, 1211. 25716111 10.1007/s00264-015-2707-9

[8] S. Clockaerts , Y. M. Bastiaansen‐Jenniskens , C. Feijt , L. De Clerck , J. A. N. Verhaar , A.‐M. Zuurmond , V. Stojanovic‐Susulic , J. Somville , M. Kloppenburg , G. J. V. M. Van Osch , Ann. Rheum. Dis. 2012, 71, 1012. 22307941 10.1136/annrheumdis-2011-200688

[9] I. R. Klein‐Wieringa , M. Kloppenburg , Y. M. Bastiaansen‐Jenniskens , E. Yusuf , J. C. Kwekkeboom , H. El‐Bannoudi , R. G. H. H. Nelissen , A. Zuurmond , V. Stojanovic‐Susulic , G. J. V. M. Van Osch , R. E. M. Toes , A. Ioan‐Facsinay , Ann. Rheum. Dis. 2011, 70, 851. 21242232 10.1136/ard.2010.140046

[10] H. Wu , M. Fu , J. Liu , W. Chong , Z. Fang , F. Du , Y. Liu , L. Shang , L. Li , Mol Cancer 2021, 20, 71. 33926452 10.1186/s12943-021-01365-z PMC8081769

[11] S. Aday , I. Hazan‐Halevy , A. Chamorro‐Jorganes , M. Anwar , M. Goldsmith , N. Beazley‐Long , S. Sahoo , N. Dogra , W. Sweaad , F. Catapano , S. Ozaki‐Tan , G. D. Angelini , P. Madeddu , A. V. Benest , D. Peer , C. Emanueli , Mole Ther 2021, 29, 2239. 10.1016/j.ymthe.2021.03.015 PMC8261169 33744469

[12] C. Théry , K. W. Witwer , E. Aikawa , M. J. Alcaraz , J. D. Anderson , R. Andriantsitohaina , A. Antoniou , T. Arab , F. Archer , G. K. Atkin‐Smith , D. C. Ayre , J.‐M. Bach , D. Bachurski , H. Baharvand , L. Balaj , S. Baldacchino , N. N. Bauer , A. A. Baxter , M. Bebawy , C. Beckham , A. Bedina Zavec , A. Benmoussa , A. C. Berardi , P. Bergese , E. Bielska , C. Blenkiron , S. Bobis‐Wozowicz , E. Boilard , W. Boireau , A. Bongiovanni , et al., J. Extracell vesicles 2018, 7, 1535750. 30637094 10.1080/20013078.2018.1535750 PMC6322352

[13] R. Garcia‐Martin , B. B. Brandao , T. Thomou , E. Altindis , C. R. Kahn , Cell Rep. 2022, 38, 110277. 35045290 10.1016/j.celrep.2021.110277 PMC8867597

[14] M. Tkach , C. Théry , Cell 2016, 164, 1226. 26967288 10.1016/j.cell.2016.01.043

[15] M. Mathieu , L. Martin‐Jaular , G. Lavieu , C. Théry , Nat. Cell Biol. 2019, 21, 9. 30602770 10.1038/s41556-018-0250-9

[16] L. Mashouri , H. Yousefi , A. R. Aref , A. M. Ahadi , F. Molaei , S. K. Alahari , Mol Cancer 2019, 18, 75. 30940145 10.1186/s12943-019-0991-5 PMC6444571

[17] D. K. Jeppesen , A. M. Fenix , J. L. Franklin , J. N. Higginbotham , Q. Zhang , L. J. Zimmerman , D. C. Liebler , J. Ping , Q. Liu , R. Evans , W. H. Fissell , J. G. Patton , L. H. Rome , D. T. Burnette , R. J. Coffey , Cell 2019, 177, 428. 30951670 10.1016/j.cell.2019.02.029 PMC6664447

[18] N. Bister , C. Pistono , B. Huremagic , J. Jolkkonen , R. Giugno , T. Malm , J. Extracell vesicles 2020, 10, e12002. 33304471 10.1002/jev2.12002 PMC7710128

[19] T. Kato , S. Miyaki , H. Ishitobi , Y. Nakamura , T. Nakasa , M. K. Lotz , M. Ochi , Arthritis Res. Ther. 2014, 16, R163. 25092378 10.1186/ar4679 PMC4261911

[20] H. Jia , L. Duan , P. Yu , Y. Zhou , R. Liu , H. Wang , Int. Immunopharmacol. 2022, 111, 109135. 35987145 10.1016/j.intimp.2022.109135

[21] S. Song , E. W.‐F. Lam , T. Tchkonia , J. L. Kirkland , Y. Sun , Trends Biochem. Sci. 2020, 45, 578. 32531228 10.1016/j.tibs.2020.03.008 PMC7649645

[22] R. F. Loeser , J. A. Collins , B. O. Diekman , Nat. Rev. Rheumat. 2016, 12, 412. 10.1038/nrrheum.2016.65 PMC4938009 27192932

[23] K. Mcculloch , G. J. Litherland , T. S. Rai , Aging Cell 2017, 16, 210. 28124466 10.1111/acel.12562 PMC5334539

[24] O. H. Jeon , N. David , J. Campisi , J. H. Elisseeff , J. Clin. Invest. 2018, 128, 1229. 29608139 10.1172/JCI95147 PMC5873863

[25] R. Lasagni Vitar , F. Triani , M. Barbariga , P. Fonteyne , P. Rama , G. Ferrari , Stem Cell Rep. 2022, 17, 849. 10.1016/j.stemcr.2022.02.012 PMC9023781 35334220

[26] A. En , Y. Takauji , K. Miki , D. Ayusawa , M. Fujii , FEBS Open Bio. 2020, 10, 237. 10.1002/2211-5463.12775 PMC6996348 31825172

[27] R. Arai , A. En , Y. Takauji , K. Maki , K. Miki , M. Fujii , D. Ayusawa , Mech Ageing Dev 2019, 178, 25. 30615890 10.1016/j.mad.2019.01.001

[28] M. Arra , G. Swarnkar , Y. Alippe , G. Mbalaviele , Y. Abu‐Amer , Bone Res. 2022, 10, 12. 35145063 10.1038/s41413-021-00183-9 PMC8831569

[29] X. Cao , P. Luo , J. Huang , C. Liang , J. He , Z. Wang , D. Shan , C. Peng , S. Wu , Stem Cell Res Ther 2019, 10, 86. 30867061 10.1186/s13287-019-1193-1 PMC6416972

[30] Z. Zhou , S. Tang , X. Nie , Y. Zhang , D. Li , Y. Zhao , Y. Cao , J. Yin , T. Chen , G. Ruan , Z. Zhu , X. Bai , W. Han , C. Ding , Inflamm Res 2021, 70, 1129. 34562102 10.1007/s00011-021-01503-9

[31] M. Colombo , G. Raposo , C. Théry , Annu Rev Cell Dev Bi 2014, 30, 255. 10.1146/annurev-cellbio-101512-122326 25288114

[32] G. Casella , R. Munk , K. M. Kim , Y. Piao , S. De , K. Abdelmohsen , M. Gorospe , Nucleic Acids Res. 2019, 47, 11476. 31612919 10.1093/nar/gkz879 PMC6868356

[33] R. W. Carthew , E. J. Sontheimer , Cell 2009, 136, 642. 19239886 10.1016/j.cell.2009.01.035 PMC2675692

[34] S. Tao , T. Yuan , Y. Zhang , W. Yin , S. Guo , C. Zhang , Theranostics 2017, 7, 180. 28042326 10.7150/thno.17133 PMC5196895

[35] L. Yu , B. Sui , W. Fan , L. Lei , L. Zhou , L. Yang , Y. Diao , Y. Zhang , Z. Li , J. Liu , X. Hao , J. Extracell vesicles 2021, 10, e12056. 33489015 10.1002/jev2.12056 PMC7812369

[36] G. Mao , Z. Zhang , S. Hu , Z. Zhang , Z. Chang , Z. Huang , W. Liao , Y. Kang , Stem Cell Res Ther 2018, 9, 247. 30257711 10.1186/s13287-018-1004-0 PMC6158854

[37] K. Lee , J. Lee , H. Kim , S. Yeom , C. Woo , Y. Jung , Y. Yun , S. Park , J. Han , E. Kim , J. Sul , J. Jung , J. Park , J. Choi , Y. Cho , D. Jo , J. Extracell vesicles 2021, 10, e12152. 34596354 10.1002/jev2.12152 PMC8485335

[38] Y. Zhuang , S. Song , D. Xiao , X. Liu , X. Han , S. Du , Y. Li , Y. He , S. Zhang , Front Mol Bio sci 2021, 8, 766115. 10.3389/fmolb.2021.766115 PMC8802296 35111808

[39] Y.‐Y. Law , W.‐F. Lee , C.‐J. Hsu , Y.‐Y. Lin , C.‐H. Tsai , C.‐C. Huang , M.‐H. Wu , C.‐H. Tang , J.‐F. Liu , Aging 2021, 13, 17227. 34198264 10.18632/aging.203201 PMC8312412

[40] J. Ni , X. Wang , S. Chen , H. Liu , Y. Wang , X. Xu , J. Cheng , J. Jia , X. Zhen , Brain, Behav., and Immun. 2015, 49, 75. 10.1016/j.bbi.2015.04.014 25934573

[41] Y. Wu , Y. Zhang , X. Zheng , F. Dai , Y. Lu , L. Dai , M. Niu , H. Guo , W. Li , X. Xue , Y. Bo , Y. Guo , J. Qin , Y. Qin , H. Liu , Y. Zhang , T. Yang , L. Li , L. Zhang , R. Hou , S. Wen , C. An , H. Li , W. Xu , W. Gao , Mol. Cancer 2020, 19, 99. 32487167 10.1186/s12943-020-01215-4 PMC7265647

[42] F. Eymard , X. Chevalier , Joint Bone Spine 2016, 83, 389. 27068617 10.1016/j.jbspin.2016.02.016

[43] M. Favero , H. El‐Hadi , E. Belluzzi , M. Granzotto , A. Porzionato , G. Sarasin , A. Rambaldo , C. Iacobellis , A. Cigolotti , C. G. Fontanella , A. Natali , R. Ramonda , P. Ruggieri , R. De Caro , R. Vettor , M. Rossato , V. Macchi , Rheumatology 2017, 56, 1784. 28957567 10.1093/rheumatology/kex287

[44] H. Onuma , K. Tsuji , T. Hoshino , K. Inomata , M. Udo , Y. Nakagawa , H. Katagiri , K. Miyatake , T. Watanabe , I. Sekiya , T. Muneta , H. Koga , J. Orthop. Res. 2020, 38, 1296. 31903621 10.1002/jor.24580

[45] A. B. Herman , C. Anerillas , S. C. Harris , R. Munk , J. L. Martindale , X. Yang , K. Mazan‐Mamczarz , Y. Zhang , I. J. Heckenbach , M. Scheibye‐Knudsen , S. De , P. Sen , K. Abdelmohsen , M. Gorospe , Nucleic Acids Res. 2021, 49, 7389. 34181735 10.1093/nar/gkab538 PMC8287953

[46] A. Chatterjee , S. Jana , S. Chatterjee , L. M. Wastall , G. Mandal , N. Nargis , H. Roy , T. A. Hughes , A. Bhattacharyya , Brit J Cancer 2019, 121, 679. 31481734 10.1038/s41416-019-0566-7 PMC6889135

