# Supplementary material for: Extracellular Vesicles in Infrapatellar Fat Pad from Osteoarthritis Patients Impair Cartilage Metabolism and Induce Senescence
Source: Adv Sci (Weinh). 2023 Nov 30;11(3):2303614. doi: 10.1002/advs.202303614 (PMC10797473; doi:10.1002/advs.202303614)
Supplement: Supplementary file 1 — Supporting Information [file ADVS-11-2303614-s001.pdf]

ADVANCED SCIENCE
Open Access

# Supporting Information

for *Adv. Sci.*, DOI 10.1002/advs.202303614

Extracellular Vesicles in Infrapatellar Fat Pad from Osteoarthritis Patients Impair Cartilage Metabolism and Induce Senescence

*Yumei Cao, Jianzhao Ruan, Jingliang Kang, Xiaoyu Nie, Weiren Lan, Guangfeng Ruan, Jia Li, Zhaohua Zhu, Weiyu Han, Su'an Tang* and Changhai Ding**

## Supplementary Materials and Methods

## Materials and methods

### Human IPFP and cartilage tissue collection

Approved by the Ethics Committee of Zhujiang Hospital, Southern Medical University (Guangzhou, China) (2021-KY-126-01), all the human IPFP, cartilage tissue and synovial fluid needed for this study were collected from patients undergoing end-stage knee OA and given total knee replacement surgery (TKA). All patients obtained a written informed consent. The inclusion criteria included: (1) 45-79 years old without any gender limitation; (2) having symptoms of OA, knee pain especially. All clinical data were reviewed before inclusion to exclude secondary OA or inflammatory joint diseases. Supplementary table S1 shows clinical characteristics of the patients. Cartilage tissue was harvested from the tibial plateau or femoral condyle, while IPFP samples were harvested from anterior knee compartment.

### Isolation and identification of sEVs

The sEVs were isolated from fresh human IPFP specimens and synovial fluid. All the IPFP tissues were rinsed twice with phosphate buffered solution (PBS) (Biosharp, China) to remove impurity and mechanically minced into small pieces sized 3-4mm$^3$. Then, maintained in Dulbecco's Modified Eagle Medium/Nutrient Mixture F-12 (DMEM/F-12) medium (Gibco Life Technology, NY), plus with 5% EV-free fetal bovine serum (FBS), cells were cultured in an incubator at 37°C with 5% $CO_2$ for 24 h. IPFP-conditioned media and synovial fluid were collected to extract sEVs using ultracentrifugation. Briefly, the supernatant was centrifuged at 3000 g first and 10,000 g following for the removement of tissue debris to get the pure supernatant. The resuspend supernatant was filtered by 0.22 mm pore filters, followed by ultracentrifugation for 120 min at 100,000 g (Hitachi, CP100WX). Pure sEVs were used fresh or resuspension in PBS before stored at -80°C. The concentration, size, and density of sEVs were measured using a nanoparticle tracking analyzer (Flow NanoAnalyzer N30E, China). The morphology was assessed by transmission electron

microscopy (JEOL JEM-1200EX, Japan). sEVs surface markers (CD63, TSG10 and HSP70) were detected by western blot analysis. For each experiment, at least three independent IPFP-sEVs were used.

**Human chondrocytes (HCs) isolation and IPFP-HCs coculture**

HCs were isolated following a previously reported method [1]. The undamaged areas of human cartilage were harvested under sterile conditions. PBS was used to wash the dissected cartilage after the elimination of connective tissue and perichondrium. Using trypsin (Gibco Life Technology, NY) to digest for 20 min. The supernatant would then be eliminated while the minced tissues be washed for three times with PBS, digested at 37℃ for 12 h with 3 times volume of type II collagenase solution (ThermoFisher, NY). Using 5 min centrifugation at 300 g to get the cell pellet from the cell suspension, all the remaining cartilage would be digested again and harvested in 24 h. Seeded in 25 $cm^2$ culture flasks containing 10% FBS (Gibco Life Technology, NY) plus with DMEM/F12, cells were cultured in a 37°C humidified atmosphere with 5% $CO_2$. For coculture experiment, IPFP tissues of OA patients were cultured in a transwell system (0.4 mm), which was placed above the primary HCs. The HCs and IPFP share the same culture medium without direct cell-cell contact. For blockade of IPFP generation of sEVs, 10-μM GW4869 (Sigma-Aldrich, USA) was applied in IPFP culture for 24 h.

**Internalization of sEVs**

The internalization of sEVs into HCs was evaluated using fluorescent microscopy. IPFP-sEVs collected from human IPFP tissues were labeled with PKH26 for 30 min and then added into HCs in a concentration-dependent manner for 24 h. To assess their internalization into chondrocytes, cells were treated with 4% paraformaldehyde (PFA) and stained with phalloidin for 30 min in dark. Finally, 4',6-Diamidino-2-phenylindole, dihydrochloride (DAPI) was used to label the nucleus for immunofluorescence. Images were taken by a fluorescent microscope (Nikon) while the analysis was performed with a Nikon image analysis software.

**RNA extraction and quantitative real-time polymerase chain reaction (qRT-PCR)**

Total HCs RNA extraction was performed using TRIzol kit (Accurate Biology, China) according to the manufacturer's instructions. To investigate the expression of miRNA and mRNA respectively, the Evo M-MLV RT Premix for qPCR (Accurate Biology, China) was used to performed reverse transcription at 42 °C for 15 minutes before inactivation at 85 °C for 5 seconds using 1000ng total RNA. Using the SYBR Green Premix Pro Taq HS qPCR Kit (Accurate Biology, China) for assessment of amplification of qRT-PCR in a CFX Connection Real-Time System (Bio-Rad, USA) followed the cycling conditions: 30 s at 95°C before 40 cycles of 95°C for 5 s and 60°C for 30 s. The internal reference U6 for miRNAs and GAPDH for mRNAs were used to normalize the reactions, which were performed in duplicate. Evaluations of relative mRNA/miRNA expression levels were assayed by using the $2^{-\triangle\triangle Ct}$ method. Primers are listed in Table S2A.

**Western blotting**

After lysed with radio-immunoprecipitation assay buffer (RIPA, Biosharp, China) plus with protease and phosphorylase inhibitor (Biyotime, China), protein concentrations of HCs were qualified using bicinchoninic acid (BCA) protein assay kit (Beyotime, China). Then the protein concentrations were separated by 10% SDS-PAGE gels before the transfer onto the polyvinylidene fluoride (PVDF) membranes (Immobilon P, Millipore), which were followed by a blockage in 10 mM Tris-buffered saline (TBS) containing 5% nonfat skimmed milk. Incubated with the primary antibodies of HSP70, TSG101, CD63 (1:1000, Abcam, Cat# ab275018), SOX9 (1:1000, Proteintech, Cat# 67439-1-lg), MMP13 (1:1000, Abcam, Cat# ab84594), MMP3 (1:1000, Abcam, Cat# ab52915), GAPDH (1:5000, Proteintech, Cat# 60004-1-Ig), LBR (1:1000, Abcam, Cat# ab32535), CBX2 (1:1000, Proteintech, Cat# 15579-1-AP) in 5% BSA dilution at 4 ℃ overnight, the membrane would then be washed with TBST before the incubation with a secondary antibody (1:5000,

ABclonal, Cat# AS014 or ABclonal, Cat# AS003) for 1 h at room temperature. After three times washes, ECL (Merck millipore) were used to detect the chemiluminescent imaging of the blots by a chemiluminescence system. The endogenous reference protein GAPDH was used as the loading control. The chemiluminescent images were captured and analyzed using a computer program (ImageJ, USA). Three separate reproducible experiments were performed for the presented blots .

**Immunofluorescence staining**

15 mm cell slides (NEST Biotechnology, China) were placed in 24-well plates to seed the HCs for immunofluorescence staining. After fixation with 4% PFA, permeabilization with 0.5% Triton X-100, blockage by 5% BSA, HCs were then incubated with primary antibody of COL2AL (1:200, Abcam, Cat# ab34712), Aggrecan (1:200, Proteintech, Cat#13880-1-AP), γ-H2AX (1:200, Abcam, Cat# ab34712), $p16^{INK4a}$ (1:200, Abcam, Cat# ab51243), LBR (1:200, Abcam, Cat# ab32535), MMP3 (1:200, Abcam, Cat# ab52915) at 4℃ overnight. Fluorescent Alexa Fluor® 555-conjugated secondary antibody (1:500, Cell Signaling Technology, Cat# 4413) was used to incubate chondrocytes the next day in dark at 37 ℃ for 1 h. Fluorescence images were obtained using Nikon Ti2-E. We quantified the total fluorescence intensity of $p16^{INK4a}$ both in cytoplasm and nucleus.

**Alcian blue and Toluidine blue staining**

HCs were seeded on 12-well plates with PBS or IPFP-sEVs stimulation for 14 days. The culture medium was changed every three days. Fixed with 4% PFA, washed with PBS, cells were then stained with alcian blue and toluidine blue dye (Solarbio, China) for 15 min, separately. Finally, the stain was aspirated and photographed with a camera.

**SA-β-Gal staining**

HCs with positive SA-β-Gal were detected using kit according to the manufacturer's instructions (Beyotime, China). Fixed with β-galactosidase staining fixative at room

temperature for 15 minutes, following three times wash with PBS, chondrocytes were incubated with SA-β-Gal staining solution at 37℃ overnight. Then, the SA-β-Gal positive chondrocytes were determined under microscopy (Leica DM2500).

**Cartilage explant culture**

Human samples from undamaged area of knee joint cartilage were collected from patients with OA and were minced into small pieces sized 3-4 $mm^3$. Mice cartilage tissue derived from the femoral head. All the explants were cultured in DMEM-F12 containing 5% FBS supplemented with PBS or IPFP-sEVs. After 14 days, explants were collected and treated with 4% PFA for 36 h. Then the explants were used for the further assessment.

**sEVs-derived miRNA microarray assay**

Five samples of OA IPFP-sEVs were processed for assay. Total RNAs extraction of IPFP-sEVs were used for miRNA sequencing. Biotechnology Corporation (China) performed the miRNA library construction as well as sequencing. Briefly, total RNA samples were extracted by mirVana™ miRNA Isolation Kit (Austin TX, US). The products of PCR, which was used to reverse transcribe and amplify the small RNAs ranging from 18 to 30 nucleotides for library construction, were sequenced using the Agilent Technogies 2100 Bioanalyzer.

**In situ hybridization (ISH)**

Treated with 4% PFA, decalcified with ethylenediaminetetraacetic acid (EDTA), the ISH of human knee tissues was performed using kit (Boster Biological Technology, China) as previous described [2]. After a prehybridization at 42℃ for 2 h, followed blockage of endogenous peroxidase and digestion using pepsin diluted with 3% citric acid, the sections were given hybridization at 42℃ overnight. The blocking solution, biotinylated mouse anti digoxin, strept avidin-biotin complex and biotinylated peroxidase were added to the sections after wash with SSC. Finally, the slides were stained with Alkaline Phosphatase (AP) and observed using a microscope after

dehydration.

**MiRNAs transfection and cell treatments**

6-well plate was used to seed HCs at density of $1.5 \times 10^5$ per well. After 48 h, the cells reached about 70% confluence. Then the let-7b-5p and let-7c-5p inhibitor, which were at the concentration of 30 nM, were transfected into cells using Lipofectamine 3000 reagents (Thermofish, USA) according to the manufacturer's instructions. At the same time, cells were cultured with IPFP-sEVs at the concentration of 20 µg. The cells needed for further experiments were collected after 48 h. A list of all miRNA sequences used are provided in Table S2B.

**Luciferase assay**

The plates with 70-80% confluence of HCs was used for the transfection using Lipofectamine 3000 (Invitrogen; Carlsbad, CA, USA). Co-transfection into each group include: a total 500 ng plasmids *LBR* 3'UTR-wt, *LBR* 3'UTR-mut, 20 nmol let-7b-5p, let-7c-5p and NC. The internal reference (firefly luciferase activities) was acted by using Luciferase Assay Reagent II (Luciferase Assay Reagent, Promega) and lysis buffer. On the other hand, Stop & Glo® Reagent (Luciferase Assay Reagent, Promega) would be used to measured Renilla luciferase activities. Every experiment would be performed replicatly in three parallel wells of a 96-well plate. Finally, the measurement of Firefly/Renilla luciferase activities would be used to determine relative luciferase activity.

**Bioinformatics analysis of mRNA–miRNA interactions**

Online databases including miRDB (http://mirdb.org/), Targetscan (http://www.targetscan.org/), miRPathDB (https://mpd.bioinf.uni-sb.de/) and starbase (http://starbase.sysu.edu.cn/) were used to predict the targets of let-7b-5p and let-7c-5p. These data were intersected with core genes downregulated in senescent transcriptome,(33) the overlapping interactions were reported as a Venn diagram

constructed using a web-based tool (*http://bioinformatics.psb.ugent.be/webtools/Venn/*).

**Animal experiments**

The approval of the animal experiments of this study was conducted from Zhujiang Hospital of Southern Medical University Ethics Committee (LAEC-2020-240). For the animal sections in Fig. 1, 12 weeks old male C57BL/6 mice, which were weighted about 25 g, were divided randomly into 3 groups include: (1) Sham ; (2) DMM + vehicle; (3) DMM + GW4869. Using multiple intra-articular injections, 10 µl of vehicle or 10 µl of GW4869 (20-µM) were injected every week after the DMM surgery. For the animal sections in Fig. 3, mice were divided randomly into 4 groups include: (1) Sham + IPFP-sEVs; (2) Sham + PBS; (3) DMM + IPFP-sEVs; (4) DMM + PBS. Using multiple intra-articular injections, 10 µl of PBS or 10 µl of IPFP-sEVs (2 µg/µl) were given into the mice every two weeks after the sham or OA-induced surgery. For the animal sections in Fig. 7, mice as previously described were divided randomly into another 4 groups: (1) Sham + IPFP-sEVs + anti NC; (2) Sham + IPFP-sEVs + anti let-7b/c-5p; (3) DMM + IPFP-sEVs + anti NC; (4) DMM + IPFP-sEVs + anti let-7b/c-5p. Each group was given 10 µl in total using intra-articular injections. To induce OA model, a surgical destabilization of the medial meniscus (DMM) surgery was performed after using amobarbital sodium (25 mg/kg) intraperitoneally for anesthetization. During the surgery, a stereomicroscope was used to observe the right knee joint through a medial capsular incision. After displacing the medial meniscus medially and resecting the medial meniscotibial ligament, the incision would be stitched, and the skin was closed. All mice were sacrificed at 12 weeks after initial surgery or the first articular injection for the right knee, which was needed to conduct a further evaluation.

**Histological and immunofluorescence analysis**

All the samples, including explants and mice joints, were fixed in 4% PFA following decalcification with EDTA. Each paraffin-embedded sample for safranin o/fast green

staining, which was performed as previously described(47), was sectioned at 4 μm. According to the OARSI guideline, both the femoral condyle and tibial plateau of the cartilage was scored separately from 0 (normal) to 6 (erosion >75% of articular surface). When it comes to synovitis grade ranking from 0 (no synovitis) to 3 (severe synovitis), the scoring criteria include synovial lining layer, degree of inflammatory infiltration and activation of resident cells/synovial stroma. As for the osteophyte scoring, different grades represent the following features; 0: no osteophytes; 1: small osteophyte, 2: medium osteophyte, 3: large and mature osteophyte. For immunofluorescence, the sections were undergoing conventional dewaxing before repaired with EDTA repair solution in 65℃ constant temperature water bath for 12-14 h. After permeation of the membrane using 0.5% Triton (Regan), blockage using 5% BSA (MCE, China), the slices were incubated with primary antibodies: LBR (1:200, Abcam, Cat# ab32535); Collagen II (1:200, Abcam, Cat# ab34712); MMP3(1:200, Abcam, Cat# ab52915); $P16^{INK4a}$ (1:200, Abcam, Cat# ab51243) overnight. After washes with PBST, the fluorescent Alexa Fluor® 555-conjugated secondary antibody (Cell Signaling Technology Cat# 4413) was used for incubation, while the nuclei were stained with DAPI. Nikon Ti2-E was used for the fluorescence images acquisition. For the quantification of proteins stained with immunofluorescence, which were located in primarily cartilage matrix localization (such as Collagen II), the cartilage matrix was selected and compared across samples. As for the cytoplasmic or cell-wide distributed proteins, the whole cell would be selected.

**Statistical analysis**

At least triplicate samples were used to report the data as the mean ± SEM or 95% CI. Unpaired two-tailed Student's t test, Mann-Whitney U test for comparisons between groups or one-way analysis of variance (ANOVA) were used for statistical analysis. All of the statistical calculations were considered statistically significant when $P < 0.05$ (two-sided). PRISM 6.0 (GraphPad Software, San Diego, CA, USA) was applied to data analysis.

## References

[1] M. Gosset, F. Berenbaum, S. Thirion,C. Jacques, *Nat protoc* **2008**, *3*, 1253-1260

[2] Y. Cao, S. Tang, X. Nie, Z. Zhou, G. Ruan, W. Han, Z. Zhu,C. Ding, *EBioMedicine* **2021**, *65*, 103283

## Supplementary data (tables, figures)

## Supplementary tables

**Supplementary Table 1.** Characteristics of knee OA patients.

| **Characteristics** | **Values** |
|---|---|
| Number of patients | 30 |
| Age, years | 66.9 ± 7.6 |
| Gender (Female), n (%) | 21 (70.0) |
| Weight, kg | 66.6 ± 11.4 |
| Height, cm | 158.4 ± 7.4 |
| Body mass index, kg/m$^2$ | 26.5 ± 3.7 |

**Supplementary Table 2.** A. Primers used for qRT-PCR.

| **Gene** | **Forward primer (5'-3')** | **Reverse primer (5'-3')** |
|---|---|---|
| U6(human) | CGCTTCGGCAGCACATATAC | TTCACGAATTTGCGTGTCAT(RT) |
| Let-7b-5p | GCGCGTGAGGTAGTAGGTTGT | AGTGCAGGGTCCGAGGTATT |
| | | GTCGTATCCAGTGCAGGGTCCGAGGTATTCGCACTGGATACG |

| | | |
|---|---|---|
| | | ACAACCAC (RT) |
| Let-7a-5p | GCGCGTGAGGTAGTAGGTTGT | AGTGCAGGGTCCGAGGTATT |
| | | GTCGTATCCAGTGCAGGGTCCGAGGTATTCGCACTGGATACGACAACTAT (RT) |
| Let-7c-5p | GCGCGTGAGGTAGTAGGTTGT | AGTGCAGGGTCCGAGGTATT |
| | | GTCGTATCCAGTGCAGGGTCCGAGGTATTCGCACTGGATACGACAACCAT (RT) |
| miR-16-5p | CGCGTAGCAGCACGTAAATA | AGTGCAGGGTCCGAGGTATT |
| | | GTCGTATCCAGTGCAGGGTCCGAGGTATTCGCACTGGATACGACCGCCAA (RT) |
| miR-21-5p | GCGCGTAGCTTATCAGACTGA | AGTGCAGGGTCCGAGGTATT |
| | | GTCGTATCCAGTGCAGGGTCCGAGGTATTCGCACTGGATACGACTCAACA (RT) |
| miR-199a-3p | GCGCGACAGTAGTCTGCACAT | AGTGCAGGGTCCGAGGTATT |
| | | GTCGTATCCAGTGCAGGGTCCGAGGTATTCGCACTGGATACGACTAACCA (RT) |
| Let-7i-5p | CGCGCGTGAGGTAGTAGTTTGT | AGTGCAGGGTCCGAGGTATT |
| | | GTCGTATCCAGTGCAGGGTCCGAGGTATTCGCACTGGATACG |

| | | |
|---|---|---|
| | | ACAACAGC (RT) |
| miR-143-3p | CGCGTGAGATGAAGCACTG | AGTGCAGGGTCCGAGGTATT |
| | | GTCGTATCCAGTGCAGGGTCCGAGGTATTCGCACTGGATACGACGAGCTA (RT) |
| miR-125b-5p | GCGACGGGTTAGGCTCTTG | AGTGCAGGGTCCGAGGTATT |
| | | GTCGTATCCAGTGCAGGGTCCGAGGTATTCGCACTGGATACGACAGCTCC (RT) |
| Let-7f-5p | CGCGCGTGAGGTAGTAGATTGT | AGTGCAGGGTCCGAGGTATT |
| | | GTCGTATCCAGTGCAGGGTCCGAGGTATTCGCACTGGATACGACAACTAT (RT) |
| GAPDH | GGAGCGAGATCCCTCCAAAAT | GGCTGTTGTCATACTTCTCATGG |
| COL2A1 | TGGACGATCAGGCGAAACC | GCTGCGGATGCTCTCAATCT |
| ACAN | ACTCTGGGTTTTCGTGACTCT | ACACTCAGCGAGTTGTCATGG |
| SOX9 | AGCGAACGCACATCAAGAC | CTGTAGGCGATCTGTTGGGG |
| MMP3 | CGGTTCCGCCTGTCTCAAG | CGCCAAAAGTGCCTGTCTT |
| MMP13 | TCCTGATGTGGGTGAATACAAT | GCCATCGTGAAGTCTGGTAAAAT |
| IL-6 | ACTCACCTCTTCAGAACGAATTG | CCATCTTTGGAAGGTTCAGGTTG |
| IL-8 | TTTTGCCAAGGAGTGCTAAAGA | AACCCTCTGCACCCAGTTTTC |
| TNF-α | CCTCTCTCTAATCAGCCCTCT | GAGGACCTGGGAGTAGATGA |

| | | |
|---|---|---|
| | G | G |
| CCL2 | CAGCCAGATGCAATCAATGCC | TGGAATCCTGAACCCACTTCT |
| MMP1 | AAAATTACACGCCAGATTTGCC | GGTGTGACATTACTCCAGAGTTG |
| MMP10 | TGCTCTGCCTATCCTCTGAGT | TCACATCCTTTTCGAGGTTGTAG |
| CDKN2A | GATCCAGGTGGGTAGAAGGTC | CCCCTGCAAACTTCGTCCT |
| CCL4 | CTGTGCTGATCCCAGTGAATC | TCAGTTCAGTTCCAGGTCATACA |
| LBR | CGAGGGAGTCGATCAAGGTCA | CTTCAGAATCAGCGGAGTCAAT |
| CBX2 | GCCCAGCACTGGACAGAAC | CACTGTGACGGTGATGAGGTT |

Abbreviation: U6, small nuclear RNA; GAPDH, glyceraldehyde-3-phosphate dehydrogenase; COL2A1, collagen type II alpha 1 chain; ACAN, aggrecan; SOX9, SRY-box transcription factor 9; MMP3, matrix metallopeptidase 3; MMP13, matrix metallopeptidase 13; IL-6, interleukin 6; IL-8, interleukin 8; TNF-α, tumour necrosis factor alpha; CCL2, C-C motif chemokine ligand 2; MMP1, matrix metallopeptidase 1; MMP10, matrix metallopeptidase 10; CDKN2A, cyclin dependent kinase inhibitor 2A; CCL4, C-C motif chemokine ligand 4; LBR, lamin B receptor; CBX2, chromobox 2.

**Supplementary Table 2.** B. Sequence of oligos.

| **Oligo** | **sense（5'-3'）** |
|---|---|
| hsa-let-7b-5p inhibitor | AACCACACAACCUACUACCUCA |
| hsa-let-7b-5p inhibitor | AACCAUACAACCUACUACCUCA |

| **Oligo** | **Sense (5’-3’)** | **Antisense (5’-3’)** |
|---|---|---|
| siLBR | GCUGAUUCUGAAGCCAUUUTT | AAAUGGCUUCAGAAUCAGCTT |

## Supplementary figures

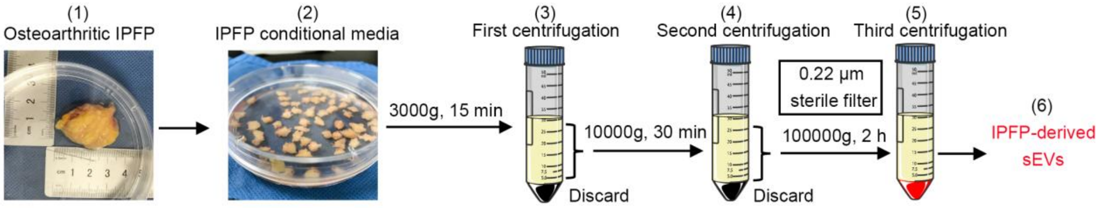


**Supplementary Figure 1.** sEVs extracted from IPFP explant-conditional medium. Schematic diagram showing the process of sEVs isolation and enrichment.

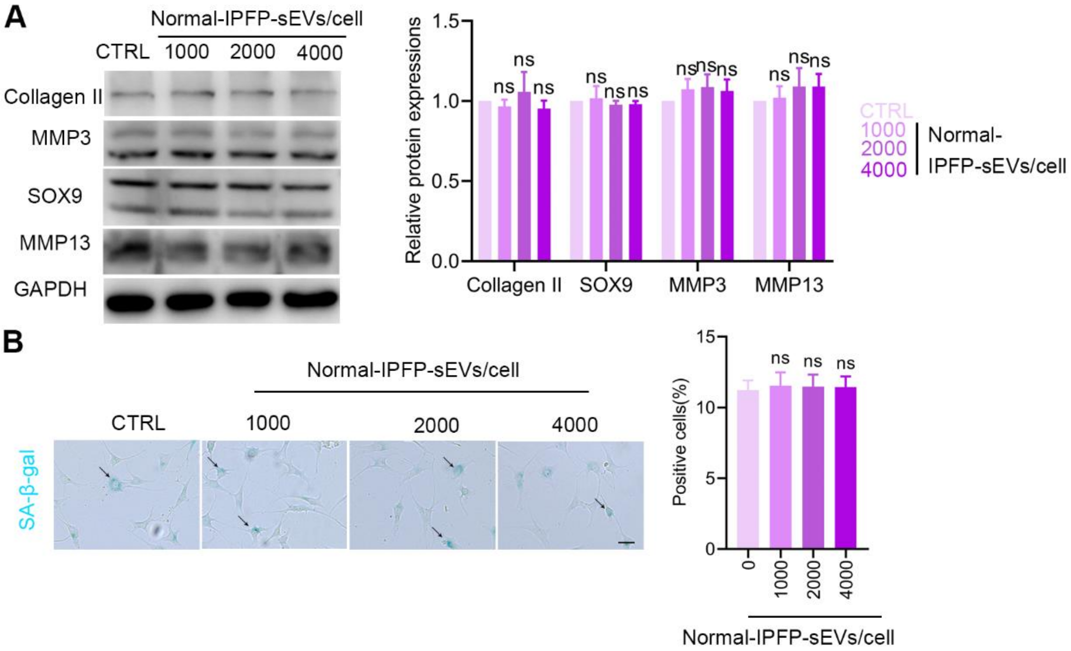


**Supplementary Figure 2**. A) Western blotting analysis of Collagen II, SOX9, MMP3 and MMP13 protein levels in chondrocytes after normal IPFP-sEVs stimulation. The data were normalized to GAPDH. B) SA-β-gal staining (left) representing the effects of normal IPFP-sEVs on senescence and SA-β-gal positive cell counting (right) of HCs. Scale bar, 100 μm. ns: no significant difference. All Data are shown as means ± SEM of three independent experiments. One-way ANOVA was used for comparison between multiple groups.

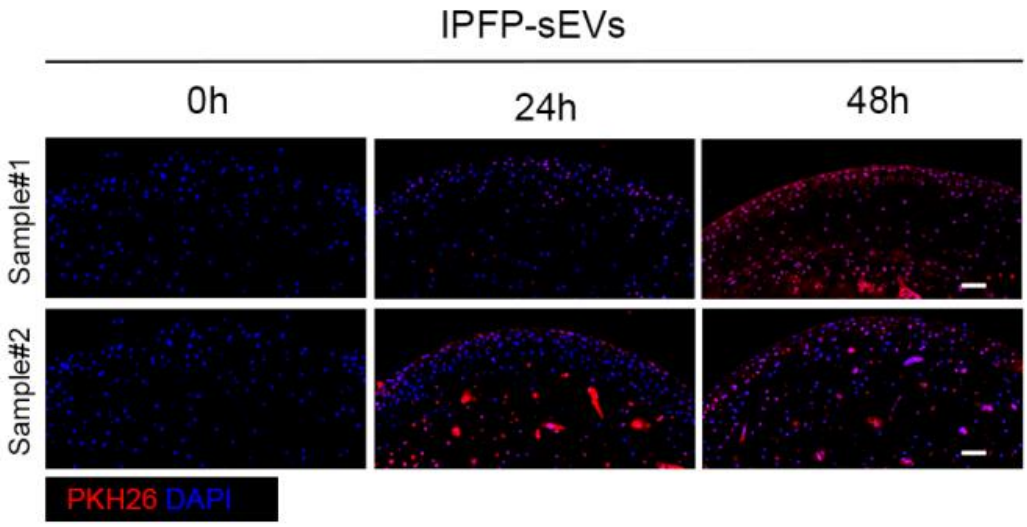


**Supplementary Figure 3.** Penetration of IPFP-sEVs in mouse femoral heads. Representative fluorescence images of cross sections of mouse femoral heads incubated with PKH26-labeled IPFP-sEVs (n=2). Scale bar, 25μm.

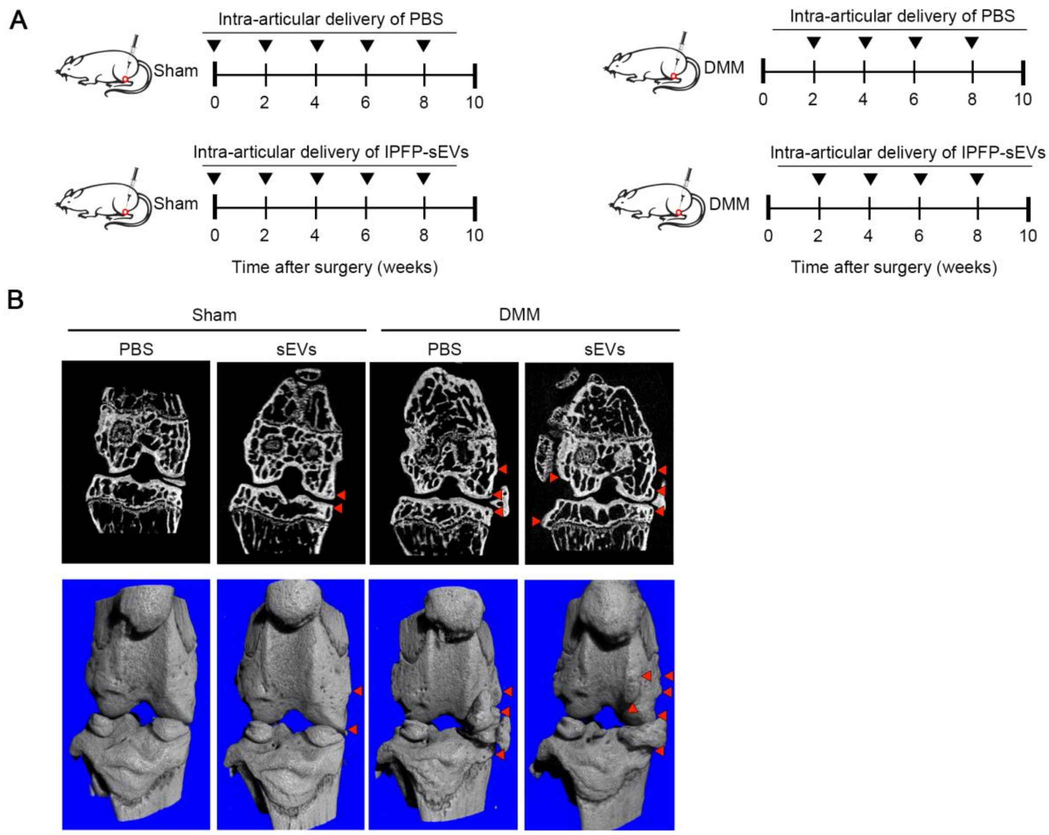


**Supplementary Figure 4.** IPFP-sEVs induce degradative effects *in vivo*. A) A flow diagram showing the time of intra-articular injection. B) Osteophytes (red arrows) were revealed by 2D (top) and 3D (bottom) micro-computed tomography (micro-CT) images.

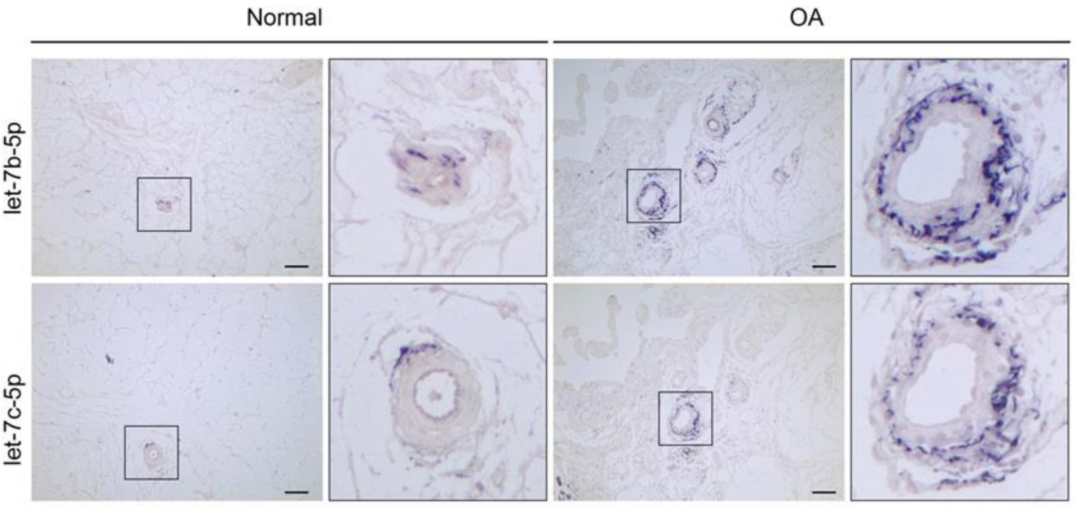


**Supplementary Figure 5**. In situ hybridization (ISH) of let-7b-5p and let-7c-5p in IPFP tissue from normal and OA patients. Scale bar, 50 µm.

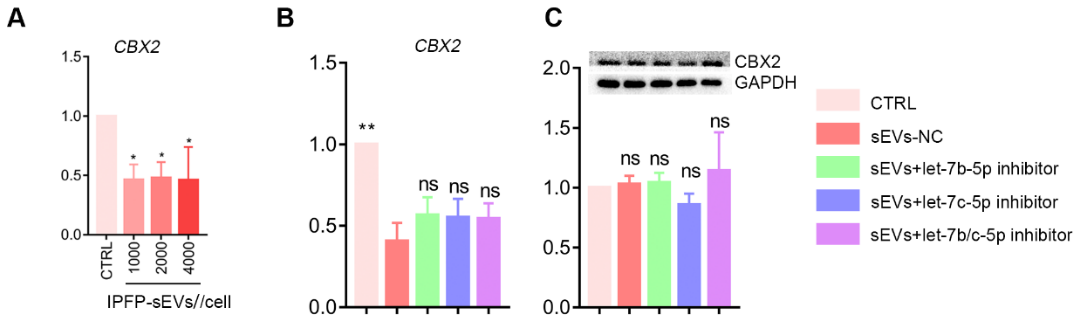


**Supplementary Figure 6.** A) qRT-PCR analysis of *CBX2* in HCs stimulated with IPFP-sEVs for 0 (control), 1000, 2000, or 4000 IPFP-sEVs/cell for 24 hours. B) mRNA expression of *CBX2* was measured by qRT-PCR in chondrocytes after IPFP stimulation or a combination of IPFP-sEVs and let-7b/c-5p inhibition. C) Western blotting analysis of CBX2 protein level in chondrocytes as treated above. The data was normalized to GAPDH. ns: no significant difference, **P*<0.05. All data are shown as means ± SEM of three independent experiments. One-way analysis of variance (ANOVA) for multiple groups.

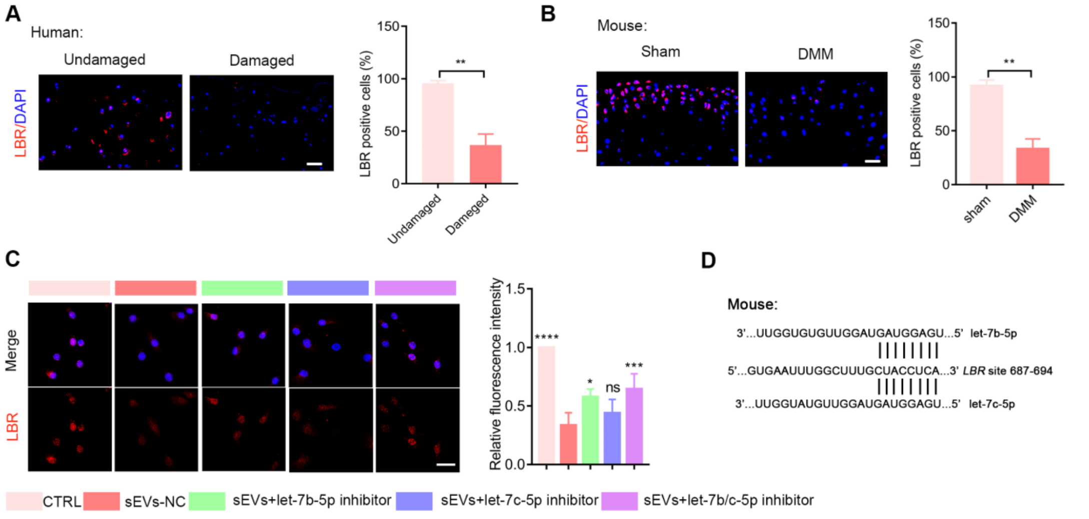


**Supplementary Figure 7.** LBR is decreased in osteoarthritic cartilage. A) Immunofluorescence analysis (left) and quantification data (right) of LBR in undamaged and damaged zones in OA cartilage. Scale bar: 100 μm. B) Immunofluorescence analysis (left) and quantification data (right) of LBR of mice cartilage in sham and DMM mouse model. Scale bar, 100 μm. C) Representative

images of LBR assayed by immunofluorescence confocal microscopy in HCs after IPFP-sEVs stimulation or a combination of IPFP-sEVs and let-7b-5p or let-7c-5p inhibition. Scale bar, 25 μm. D) Schematic representation of a predicted binding site of let-7b-5p and let-7c-5p in the 3′UTR of *LBR* mRNA in mice. ns: no significant difference, **P*<0.05, ***P*<0.01, ****P*<0.001, *****P*<0.0001. All data are shown as means ± SEM of three independent experiments in (A), (B) and (C). Student's *t* test was used for comparison between two groups and one-way analysis of variance (ANOVA) for multiple groups.

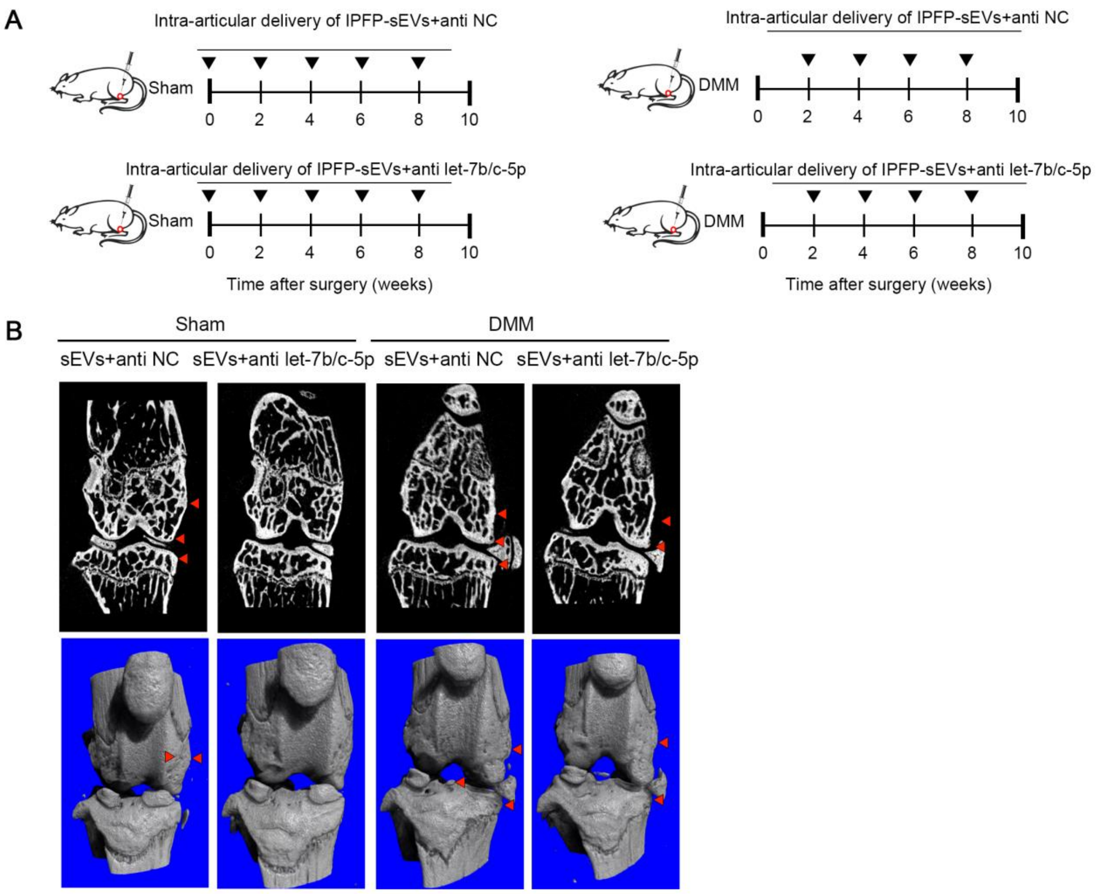


**Supplementary Figure 8.** Intra-articular injection of antagomir-let-7b-5p and let-7c-5p demolishes the deteriorated effect of IPFP-sEVs on knee joint *in vivo*. A) A flow diagram showing the time of IA injection. B) Osteophytes (red arrows) were revealed by 2D (top) and 3D (bottom) micro-computed tomography (micro-CT) images.
